# A Bimodal Approach to Broadband Vibration Energy Harvesting Using Hybrid Piezoelectric–Electromagnetic Transduction

**DOI:** 10.3390/mi17050553

**Published:** 2026-04-29

**Authors:** Guangye Jia, Qiang Zhou, Huayang Zhao

**Affiliations:** 1 Shandong Provincial Key Laboratory of New Power Distribution & Utilization Technology and Equipment, Shandong University of Technology, Zibo 255000, China; 23504040590@stumail.sdut.edu.cn (G.J.); zhao4829264@163.com (H.Z.); 2 School of Electrical and Electronic Engineering, Shandong University of Technology, Zibo 255000, China

**Keywords:** vibration energy harvesting, piezoelectric–electromagnetic coupling, equivalent nonlinear spring-damping, bimodal, potential well

## Abstract

To address the issue of traditional bistable vibration energy harvesters (BVEHs) being prone to becoming trapped in a single potential well—which results in a narrowed energy harvesting bandwidth and reduced efficiency—this paper proposes a method that utilizes the nonlinear electromagnetic force generated during the induction process to modulate the kinematic behavior of the oscillator. The characteristics and influencing factors of the nonlinear force produced during electromagnetic induction are analyzed. A dual-cantilever beam structure is designed, with an iron-core coil and a magnet placed at the respective free ends. A mathematical model of a piezoelectric–electromagnetic coupled bimodal broadband vibration energy harvester is established and numerically simulated. Furthermore, a vertical vibration experimental platform is constructed to conduct frequency sweep tests. The experimental results demonstrate that the proposed piezoelectric–electromagnetic coupled bimodal broadband vibration energy harvester effectively improves energy harvesting efficiency. Within the frequency range of 5–20 Hz, the system exhibits two vibration modes, with resonant frequencies of approximately 7.7 Hz and 15.7 Hz. For a single-layer PVDF piezoelectric film, the maximum output power at the first and second resonance points is 8.9 μW and 9.7 μW, respectively. The electromagnetic module achieves maximum output powers of 0.39 W and 0.71 W. Moreover, within the frequency ranges of 6.3–9.8 Hz and 14–17.7 Hz (a total bandwidth of 7.2 Hz), the device maintains a stable power output. The effective bandwidth is broadened by approximately 80%, demonstrating excellent broadband performance.

## 1. Introduction

With the rapid development of Internet of Things (IoT) technology, a large number of sensor nodes used for environmental monitoring, structural health monitoring, and related fields are distributed in various environments [1,2]. To satisfy the power supply requirements of these distributed devices, ambient energy harvesting technology—which scavenges renewable energy sources such as solar, thermal, and vibration energy from the environment [3,4] to provide continuous and stable power for low-power electronic devices—has emerged as an active research area [5].

Among various ambient energy sources, vibration energy [6] has attracted considerable attention due to its ubiquitous availability, relatively high energy density, and independence from lighting conditions. Environmental vibration sources are diverse, encompassing machinery operation, human motion, structural vibrations of bridges, and wind-induced oscillations. By utilizing transduction mechanisms such as the piezoelectric effect and electromagnetic induction [7,8,9], ambient vibrational energy can be converted into electrical energy to power wireless sensor network nodes [10], thereby enabling self-powered operation of sensors, which is of great significance for promoting the development of distributed passive sensor networks. However, numerous challenges persist in the practical harvesting of environmental vibration energy. Vibration sources are characterized by low frequencies, wide bandwidths, and strong randomness. Conventional methods utilizing linear cantilever beams combined with piezoelectric energy harvesting [11] struggle to adapt to external environmental vibration frequencies, resulting in low energy conversion efficiency and limited applicability for powering micro-sensors. Extensive research has been conducted by scholars worldwide [12] to address these limitations, among which introducing nonlinear forces (such as magnetic forces and nonlinear elastic restoring forces) to broaden the vibration bandwidth of cantilever beams represents one of the most effective approaches. Andò et al. proposed a vibration energy harvesting method based on bistable buckled beams [13], utilizing the prestress of elastic beams to achieve wideband mechanical vibration energy harvesting. However, when external excitation is insufficient, the potential barrier formed by the prestress of the elastic beam forces the device to become trapped within a single potential well, undergoing small-amplitude oscillations that significantly diminish energy harvesting performance. Lan and Qin proposed an improved method to reduce the potential barrier height [14] by introducing an additional small magnet between two fixed magnets in a bistable magnetically coupled structure. By utilizing its attractive force to decrease the potential energy barrier, this approach significantly lowers the excitation threshold for inter-well jumping while maintaining the bistability of the system. Numerous similar methods for lowering the potential barrier have been proposed: Zhou et al. presented a flexible bistable harvesting strategy with tunable potential energy functions [15], in which fixed magnets were replaced by movable magnets mounted on clamped-clamped elastic beams, utilizing magnetic repulsion to dynamically adjust the flexural deformation of the clamped beams. Jackson et al. [16] designed three magnetic coupling configurations—namely in-plane single magnet, in-plane double magnets, and out-of-plane arrangements—employing magnetic repulsion and attraction to modulate system stiffness and amplitude, respectively, thereby effectively broadening the operational bandwidth under low acceleration environments below 0.1 g. Zhang et al. [17] proposed a linear-arched composite beam tristable harvester, achieving transitions among mono-, bi-, and tristable states by adjusting magnet spacing to reduce potential barrier height, obtaining large-amplitude responses under low-frequency excitation. Chen et al. [18] introduced externally connected elastic magnets to design a curved bistable harvester with variable potential wells, where springs adaptively regulated the barrier height to facilitate inter-well jumping at low excitation levels, improving the effective bandwidth by 37% compared to conventional structures. Shao et al. proposed a magnetically coupled piezoelectric dual-beam energy harvesting method [19], which lowered the potential barrier through magnetic coupling, achieving large-amplitude motion under weak excitation conditions and broadening the energy harvesting bandwidth while enhancing output performance. Atmeh et al. [20] constructed a magnetically coupled dual-cantilever frequency up-conversion harvester, wherein magnetic repulsion coupling between a low-frequency beam and a high-frequency piezoelectric beam converted low-frequency excitation into high-frequency response while simultaneously reducing the potential barrier. Inspired by the flapping motion of seagull wings, Wu et al. [21] designed an S-shaped bistable piezoelectric harvester, which achieved more uniform stress distribution through the bionic configuration, reducing stress concentration by 41.2% compared to traditional cantilever beams, and delivered a peak power of 0.734 mW at 18 Hz. Bradai et al. [22] further explored bistable laminated plate structures, utilizing snap-through deformation of glass fiber reinforced polymer (GFRP) laminated plates to drive piezoelectric patches, and enhanced energy harvesting efficiency by optimizing patch orientations (concentric/radial) and mass distribution. Jin Hong et al. proposed a bistable piezoelectric energy harvesting method utilizing magnetic attraction and repulsion between upper and lower beams [23], achieving mutual excitation and collaborative bandwidth broadening between the upper and lower cantilever beams, thereby enhancing both the bandwidth and output power of vibration energy harvesting.

Other scholars have also worked to improve energy harvesting efficiency by coupling multiple power generation methods on the basis of introducing nonlinear forces. Fan et al. [24] proposed a bidirectional hybrid energy harvesting method that achieves high-efficiency collection under low-frequency mechanical vibrations (<10 Hz) through a piezoelectric–electromagnetic composite transduction mechanism coupled with a levitated magnet structure. Zhang et al. [25] presented an energy harvesting method based on the coupling of geometric and electromagnetic nonlinearities, using a rigid linkage mechanism to couple magnet motion with cantilever beam deformation, achieving high-efficiency unidirectional energy collection within a 5–13 Hz range. Wang et al. [26] proposed a piezoelectric–electromagnetic hybrid harvester based on a magnetically coupled bistable M-shaped structure; by introducing repulsive magnetic coupling to lower the potential barrier height, the system achieves inter-well transitions under low external excitation, broadening the bandwidth while enhancing efficiency through electromagnetic induction. Xu et al. [27] proposed a bistable-superimposed hybrid harvesting method that couples a buckled-beam piezoelectric bistable structure with a magnetic levitation electromagnetic bistable structure to construct an enhanced hybrid harvester, realizing the amplification and efficient conversion of low-frequency vibrations. Their results showed that this structure significantly reduces the excitation threshold, widens the operating bandwidth, and improves output performance (with a peak increase of over 180%).

Zhang et al. [28] investigated a tri-stable piezoelectric–electromagnetic hybrid harvesting method under narrowband random excitation, establishing and solving an electromechanical coupled stochastic dynamic model. They constructed a tri-stable hybrid vibration energy harvester (HVEH) consisting of a piezoelectric cantilever layer, an end magnet, and opposing permanent magnets/coils, utilizing magnetic control to achieve multi-stable potential wells. The results indicated that this method enables inter-well oscillations under random excitation, thereby broadening the bandwidth and increasing average output power. Zhang et al. [29] combined the harmonic balance method with the separation of variables to construct a quad-stable piezoelectric–electromagnetic hybrid harvester. The system exhibits complex multi-solution behavior, allowing it to maintain high power output even under low input amplitudes or low-frequency conditions. Bowen Yang et al. [30] proposed a piezoelectric–electromagnetic smart structure (SS-PEH) for wave energy harvesting; based on a piezoelectric cantilever with a proof mass, a rigid-body collision mechanism was used to adjust the natural frequency of the piezoelectric unit to match external wave frequencies. Zhang X et al. [31] proposed a new hybrid harvester and conducted an in-depth study using a combination of electromechanical coupled dynamic modeling, numerical simulation, and experimental verification. The core structure consists of a piezoelectric cantilever, an end coil, and opposing fixed magnets. The results showed that by matching the optimal initial magnet spacing and load resistance, system performance was significantly improved; under excitation conditions of 16.3 Hz and 0.8 g, the total output power reached 38.2 mW, representing substantial increases of 164.6% and 60.5% compared to standalone piezoelectric or electromagnetic harvesters, respectively. Li Zhao et al. [32] developed a TMHEH hybrid energy harvester integrating piezoelectric and electromagnetic mechanisms, which dynamically reduces system potential barriers by introducing symmetric stiffness through magnetic coupling between the main and side beams. This design effectively addresses the bottleneck where traditional bi-stable structures (BEH) struggle to oscillate under weak excitation, achieving a significantly stronger dynamic response. Experimental results confirm that the peak power of its individual units reaches 6.7, 2.0, and 51.8 mW, and the system can charge a 100 µF capacitor to 22.0 V within 300 s when units are connected in parallel, offering a new path for high-efficiency energy capture in low-frequency and low-intensity environments.

In addition, recent progress in the field of electromagnetic energy harvesting has shown that the configuration of magnet arrays plays a crucial role in output performance. Furthermore, Li et al. [33] designed an electromagnetic vibration energy harvester with a replaceable alternating-polarity magnet array. The device consists of a magnet array, a dual-coil array, a magnet holder, springs, and limit structures, and is intended to enhance electromagnetic induction by creating abrupt changes in magnetic flux density along the direction of motion, thereby improving energy harvesting performance. Li et al. [34] also developed an electromagnetic energy harvester for translational vibration energy harvesting, mainly composed of a magnet array, a six-unit coil set, springs, a magnet sliding mechanism, and a housing, and systematically compared the performance of cubic and triangular magnets under Halbach and alternating-polarity configurations. The results showed that both the geometry and arrangement of the magnet array significantly affect the magnetic flux density distribution and its abrupt variations. In particular, the cubic alternating-polarity array produced magnetic flux density changes with larger magnitude and steeper transitions, thereby achieving the best output performance. Under an excitation of 1.0 g, the open-circuit peak-to-peak voltage reached 20 V, the maximum RMS power was about 35.5 mW, and the volumetric and gravimetric power densities reached 0.4955 mW/cm^3^ and 0.28 mW/g, respectively, which were clearly superior to those of the other configurations. These results demonstrate that an alternating-polarity cubic magnet array is an effective approach for enhancing the output capability of electromagnetic energy harvesters.

This paper proposes a vibration energy harvesting method that utilizes electromagnetic nonlinear forces to construct a bimodal response. The core innovation lies in using the electromagnetic nonlinear forces generated during induction to modulate the kinematic behavior of the oscillator. The advantages of this approach are twofold: the piezoelectric–electromagnetic coupling improves energy harvesting efficiency while simultaneously broadening the operating bandwidth. Compared to bistable energy harvesting methods, the bimodal approach avoids the reduction in efficiency caused by the oscillator becoming trapped in a single potential well due to potential barriers. Furthermore, compared to complex magnet array designs, the device presented in this paper is smaller in volume, simpler in structure, and lower in cost.

## 2. Structure and Working Principle of the Bimodal Piezoelectric–Electromagnetic Broadband Vibration Energy Harvester

### 2.1. Structural Design of the Bimodal Harvester

**Figure 1:**
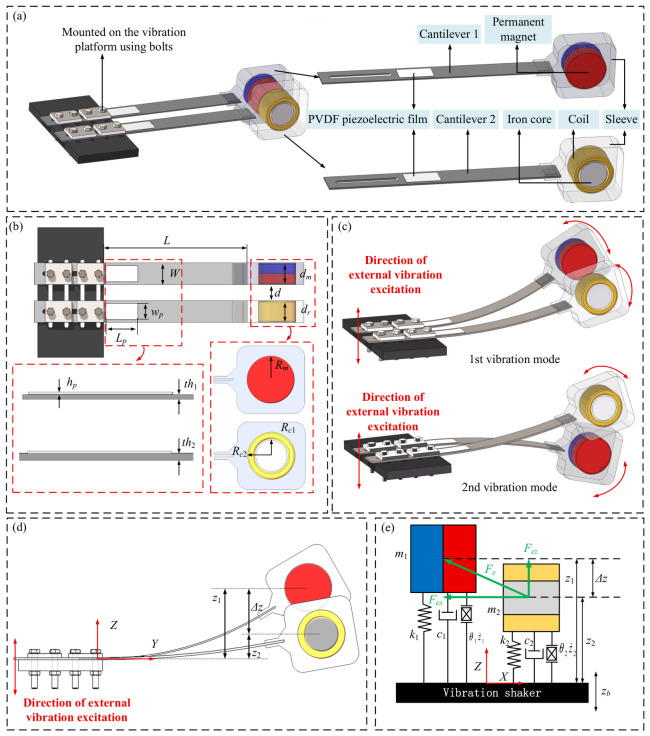
Schematic diagram and mathematical model of the piezoelectric–electromagnetic coupled bimodal vibration energy harvester: (**a**) Composition and connection methods of the device; (**b**) Structural parameters of the device; (**c**) Vibration modes of the device under external excitation; (**d**) Representation of the tip vibration displacement of the cantilever beam in the YOZ plane; (**e**) Equivalent mathematical model of the device. Color explanation: red indicates the N pole of the magnet, blue indicates the S pole of the magnet, yellow indicates the coil, and black indicates the plane of the vibration shaker.

This paper utilizes the characteristic difference in tip displacement responses at various vibration frequencies of a dual-cantilever beam structure with a specific thickness offset. By integrating the property that the relative motion between a magnet and an iron-core coil generates both electrical energy and nonlinear forces, a piezoelectric–electromagnetic coupled bimodal broadband vibration energy harvester is designed, as shown in Figure 1. The device is primarily composed of cantilever beams, piezoelectric patches, a sleeve, permanent magnets, and iron-core coils; for better visualization, the sleeve is rendered translucent. Figure 1a illustrates the components and assembly of the piezoelectric–electromagnetic coupled bimodal broadband vibration energy harvester. Figure 1b displays the structural dimensions of the key components, and Figure 1c presents the two vibration modes exhibited by the device under external vibration excitation. Specifically, Vibration Mode 1 corresponds to the mode where Cantilever 1 exhibits a large amplitude response while Cantilever 2 exhibits a small amplitude response; Vibration Mode 2 corresponds to the case where Cantilever 1 exhibits a small amplitude response and Cantilever 2 exhibits a large amplitude response.

### 2.2. Mathematical Modeling of the Bimodal Harvester

As shown in Figure 1d, let the mass per unit length of the piezoelectric cantilever be *ρS_i_*, where *ρ* is the density of the piezoelectric cantilever and *S_i_* is the cross-sectional area of piezoelectric cantilever *i*. The lengths of the cantilever beam and the piezoelectric film are *L* and *L_p_*, respectively. The Z -direction displacement at any point on cantilever *i* is denoted as *ω_i_*(*y*,*t*). The magnet and the iron-core coil at the free ends are, respectively, equivalent to lumped masses *M_t_*_1_ and *M_t_*_2_ concentrated at the tips of the cantilevers, neglecting the moment of inertia. Furthermore, *F_ex_* can be approximated as equal to the stiffness force along the width direction of the cantilever, and the cantilever primarily exhibits vibration along the *Z*-axis. The kinetic energy generated by the two piezoelectric cantilevers during the vibration process is:

(1) $$T=\sum_{i=1}^{2}\left[\frac{1}{2}\int_{0}^{L}\rho_{i}S_{i}{\left(\frac{\partial \omega_{i}(y,t)}{\partial t}\right)}^{2}dy+\frac{1}{2}M_{ti}{\left(\frac{\partial \omega_{i}(L,t)}{\partial t}\right)}^{2}\right]$$

*F_ez_* consists of two components derived from distinct physical mechanisms: the magnetic attraction component *F_kz_* (N) and the electromagnetic damping component *F_cz_* (N). *F_kz_* refers to the attractive force generated by the magnetic field between the magnet and the iron core, which causes magnetic field distortion, stores a portion of the magnetic field energy, and results in a nonlinear spring effect. *F_cz_* represents the dynamic damping force generated by electromagnetic induction during the relative motion between the coil and the magnet, manifesting as a dissipative force. In practice, *F_kz_* and *F_cz_* are not mutually independent. During the relative motion of the coil and magnet, the magnetic field within the iron core is actually a superposition of the field produced by the induced current in the coil and the field produced by the magnet; thus, *F_kz_* and *F_cz_* influence each other. Consequently, the potential energy of the system can be obtained. It is primarily composed of the potential energy generated by the bending strain of the piezoelectric cantilever beam and the magnetic potential energy formed between the iron core and the magnet. The system’s potential energy is:

(2) $$U=\int_{0}^{\Delta z}F_{kz}d\xi +\frac{1}{2}\int_{0}^{L}E_{y}I_{1}{\left(\frac{\partial^{2}\omega (y,t)}{\partial y^{2}}\right)}^{2}dy+\frac{1}{2}\int_{0}^{L}E_{y}I_{2}{\left(\frac{\partial^{2}\omega (y,t)}{\partial y^{2}}\right)}^{2}dy$$

In the equation, *E_y_* and *I_i_* represent the Young’s modulus and the area moment of inertia of the piezoelectric cantilever’s cross-section, respectively. The constitutive equations for the piezoelectric cantilever beam are expressed as follows:

(3) $$\left[\begin{array}{c} \sigma_{p}\\ D \end{array}\right]=\left[\begin{array}{cc} c_{11}^{E} & e_{31}\\ e_{31} & \varepsilon_{33}^{S} \end{array}\right]\left[\begin{array}{c} \varepsilon_{p}\\ E \end{array}\right]$$

In the equation, *c^E^*_11_ is the stiffness coefficient of the piezoelectric material, *D* is the electric displacement, *ε^S^*_33_ is the permittivity under constant stress conditions, *e*_31_ is the piezoelectric constant under constant electric field conditions, and *E* is the electric field strength. By combining the constitutive equations of the piezoelectric cantilever, the electrical potential energy of the piezoelectric material can be obtained as:

(4) $$W_{e}=\frac{1}{2}\int_{V_{p}}E_{1}D_{1}dV_{p}+\frac{1}{2}\int_{V_{p}}E_{2}D_{2}dV_{p}=\sum_{i=1}^{2}\frac{1}{2}C_{pi}v_{i}^{2}(t)$$

where *C_pi_* is the equivalent capacitance of the piezoelectric film on cantilever *i*, and *v_i_* is the voltage between the two electrodes of the piezoelectric film on cantilever *i*. The total dissipated energy of the system is expressed as:

(5) $$W_{diss}(t)=\int_{0}^{t}\left\{\sum_{i=1}^{2}\left[\int_{0}^{L}c_{vi}(y){\left(\frac{\partial \omega_{i}(y,\tau )}{\partial \tau }\right)}^{2}dy+\frac{v_{i}^{2}(\tau )}{R_{pi}}\right]+F_{\mathrm{cz}}(\Delta \dot{z}(\tau ))\Delta \dot{z}(\tau )\right\}d\tau$$

The total dissipated energy of the system comprises the energy consumed by the mechanical damping of cantilever *i* and the piezoelectric film (with damping coefficient *c_vi_*), the external resistance *R_pi_* connected to the piezoelectric film on cantilever *i*, and the nonlinear electromagnetic damping force *F_cz_*.

The work done by the system’s external excitation force is:

(6) $$W_{ext}(t)=\int_{0}^{t}\sum_{i=1}^{2}\left[-\int_{0}^{L}\rho_{i}S_{i}{\ddot{z}}_{b}(\tau )\frac{\partial \omega_{i}(y,\tau )}{\partial \tau }dy-M_{ti}{\ddot{z}}_{b}(\tau )\frac{\partial \omega_{i}(L,\tau )}{\partial \tau }\right]d\tau$$

In the equation, *z_b_*(*t*) represents the displacement of the vibration table relative to the ground, and ${\ddot{z}}_{b}$(*t*) is the excitation acceleration of the vibration table. The global vibration energy conservation equation is expressed as:

(7) $$W_{ext}=\Delta T+\Delta U+\Delta W_{ie}+W_{diss}$$

According to the Rayleigh-Ritz method, the vertical bending displacement of piezoelectric cantilever *i* can be expressed as:

(8) $$\omega (y,t)=\sum_{j=1}^{n_{r}}\phi_{ij}(y)q_{j}(t)=\phi_{i}(y)q_{j}(t)$$

where *ϕ_ij_*(*y*) is the bending mode shape function of the cantilever beam, *q_i_*(*t*) represents the time-dependent *i*-th order generalized coordinate, and *n_r_* denotes the number of mode shapes. Since the piezoelectric cantilever exhibits the same mode shape in both its first and second modes, *n_r_* = 1 is used. By substituting Equation (8) into the respective energy expressions, the lumped-parameter energy expressions are obtained as follows:

(9) $$T=\sum_{i=1}^{2}\left[\frac{1}{2}\int_{0}^{L}\rho_{i}S_{i}\phi_{i}^{2}(y)dy+\frac{1}{2}M_{ti}\phi_{i}^{2}(L)\right]{\dot{q}}_{i}^{2}(t)=\frac{1}{2}M_{1}{\dot{q}}_{1}^{2}+\frac{1}{2}M_{2}{\dot{q}}_{2}^{2}$$

where *M_i_* is defined as the sum of the distributed mass of the cantilever beam and the mass of the tip magnet or iron-core coil (also referred to as the equivalent mass).

(10) $$M_{i}=\int_{0}^{L}\rho_{i}S_{i}\phi_{i}^{2}(y)dy+M_{ti}\phi_{i}^{2}(L)$$

The potential energy is expressed as:

(11) $$\begin{array}{l} U=\sum_{i=1}^{2}\left[\frac{1}{2}\int_{0}^{L}E_{y}I_{i}{\left({\ddot{\phi }}_{i}(y)\right)}^{2}dy\right]q_{i}^{2}(t)+\int_{0}^{\phi_{1}(L)q_{1}(t)-\phi_{2}(L)q_{2}(t)}F_{kz}(\zeta )d\zeta \\ =\frac{1}{2}K_{1}q_{1}^{2}+\frac{1}{2}K_{2}q_{2}^{2}+\int_{0}^{\phi_{1}(L)q_{1}(t)-\phi_{2}(L)q_{2}(t)}F_{kz}(\zeta )d\zeta \end{array}$$

where *K_i_* is the equivalent stiffness of the piezoelectric cantilever beam, which is expressed as:

(12) $$K_{i}=\int_{0}^{L}E_{y}I_{i}{\left({\ddot{\phi }}_{i}(y)\right)}^{2}dy$$

Similarly, the energy dissipated by the system and the equivalent mechanical damping of the piezoelectric cantilever beam are, respectively, expressed as:

(13) $$W_{diss}(t)=\int_{0}^{t}\left[c_{1}{\dot{q}}_{1}^{2}(\tau )+c_{2}{\dot{q}}_{2}^{2}(\tau )+\frac{v_{1}^{2}(\tau )}{R_{p1}}+\frac{v_{2}^{2}(\tau )}{R_{p2}}+F_{\mathrm{cz}}(\Delta \dot{z})\Delta \dot{z}(\tau )\right]d\tau$$

(14) $$c_{i}=\int_{0}^{L}c_{vi}(y)\phi_{i}^{2}(y)dy$$

where *c_vi_*(*y*) denotes the distributed viscous damping function per unit length of the piezoelectric cantilever beam. The work done by the external excitation force is expressed as:

(15) $$W_{ext}(t)=-\int_{0}^{t}{\ddot{z}}_{b}(\tau )\left[\Gamma_{1}{\dot{q}}_{1}(\tau )+\Gamma_{2}{\dot{q}}_{2}(\tau )\right]d\tau$$

where *Γ_i_* is the sum of the distributed mass of the cantilever beam and the mass of the tip magnet or iron-core coil, as expressed in the following equation:

(16) $$\Gamma_{i}=\int_{0}^{L}\rho_{i}S_{i}\phi_{i}(y)dy+M_{ti}\phi_{i}(L)$$

Substituting Equations (8)–(16) into Equation (7) yields the electromechanical coupling model of the piezoelectric cantilever beam under the action of nonlinear forces *F_kz_* and *F_cz_*:

(17) $$\left\{\begin{array}{l} M_{1}{\ddot{q}}_{1}(t)+c_{1}{\dot{q}}_{1}(t)+K_{1}q_{1}(t)-\theta_{1}v_{1}(t)+\phi_{1}(L)\left[F_{kz}(\Delta z)+F_{cz}(\Delta \dot{z})\right]=-\Gamma_{1}{\ddot{z}}_{b}(t)\\ C_{p1}{\dot{v}}_{1}(t)+\frac{v_{1}(t)}{R_{p1}}+\theta_{1}{\dot{q}}_{1}(t)=0\\ M_{2}{\ddot{q}}_{2}(t)+c_{2}{\dot{q}}_{2}(t)+K_{2}q_{2}(t)-\theta_{2}v_{2}(t)-\phi_{2}(L)\left[F_{kz}(\Delta z)+F_{cz}(\Delta \dot{z})\right]=-\Gamma_{2}{\ddot{z}}_{b}(t)\\ C_{p2}{\dot{v}}_{2}(t)+\frac{v_{2}(t)}{R_{p2}}+\theta_{2}{\dot{q}}_{2}(t)=0 \end{array}\right.$$

where *θ_i_* is the electromechanical coupling coefficient of cantilever *i*, and its expression is given by Equation (18) below:

(18) $$\theta_{i}=\int_{V_{p}}q(t){\ddot{\phi }}_{i}e_{31}\nabla u_{i}dV_{p}$$

where ▽*u_i_* is the gradient of the electric potential distribution function of piezoelectric film *i* in the thickness direction. Furthermore, the mode shapes of the cantilever beam are identical in both vibration modes, with the tip of the beam being the point of maximum displacement. In this case, the cantilever beam with a tip mass can be equivalent to a spring–mass–damper system. By approximating *q_i_*(*t*) = *z_i_*(*t*), the electromechanical coupling model can be expressed as:

(19) $$\left\{\begin{array}{l} M_{1}{\ddot{z}}_{1}(t)+c_{1}{\dot{z}}_{1}(t)+K_{1}z_{1}(t)-\theta_{1}v_{1}(t)+F_{ez}=-M_{1}{\ddot{z}}_{b}(t)\\ C_{p1}{\dot{v}}_{1}(t)+\frac{v_{1}(t)}{R_{p1}}+\theta_{1}{\dot{z}}_{1}(t)=0\\ M_{2}{\ddot{z}}_{2}(t)+c_{2}{\dot{z}}_{2}(t)+K_{2}z_{2}(t)-\theta_{2}v_{2}(t)-F_{ez}=-M_{2}{\ddot{z}}_{b}(t)\\ C_{p2}{\dot{v}}_{2}(t)+\frac{v_{2}(t)}{R_{p2}}+\theta_{2}{\dot{z}}_{2}(t)=0 \end{array}\right.$$

This is equivalent to the mathematical model shown in Figure 1e, where the two cantilever beams and their respective tip masses are modeled as first-order forced vibration systems. The first system has a mass *M*_1_ (kg), stiffness coefficient *K*_1_ (N/m), and damping coefficient *c*_1_ (N·s/m), while the second system has a mass *M*_2_ (kg), stiffness coefficient *K*_2_ (N/m), and damping coefficient *c*_2_ (N·s/m). Due to the different stiffness coefficients and masses of the two first-order vibration systems, a relative displacement Δ*z* (mm) occurs between the two proof masses. This relative displacement induces a vertical nonlinear interaction force, *F_ez_* (N).

To further identify the factors influencing the device’s vibration displacement and output characteristics, a detailed analysis of the nonlinear interaction force *F_ez_* is required. As established in the previous analysis, *F_ez_* can be decomposed into an attractive force component *F_kz_*, which stores magnetic field energy, and a damping force component *F_cz_*, which generates electromagnetic damping. Considering that both the initial air gap *d* and the relative displacement of the iron core fall within the near-field range, traditional equivalent magnetic dipole methods struggle to ensure sufficient accuracy. Therefore, this paper employs the Distributed Magnetic Charge Model to precisely compute the spatial magnetic field of the permanent magnet.

**Figure 2:**
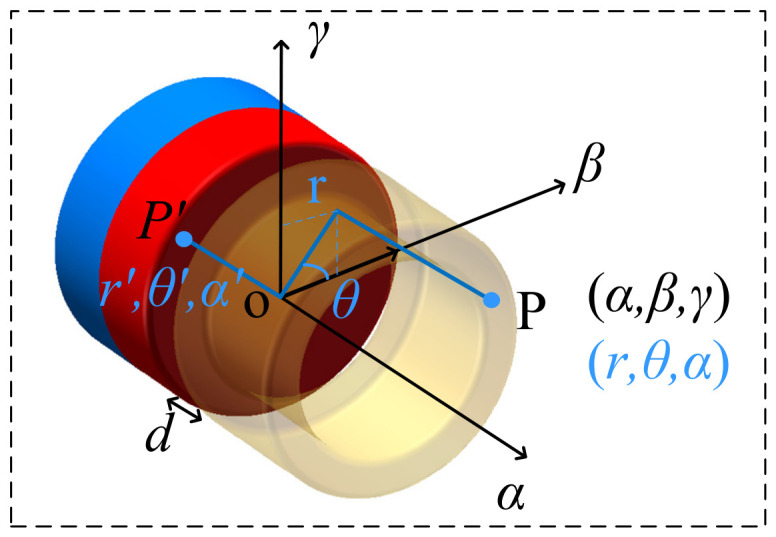
Cartesian and polar coordinate systems established with the permanent magnet as the reference.

In a system comprising a permanent magnet and an iron-core coil, a coordinate system is established with the permanent magnet acting as the reference, as illustrated in Figure 2. Under the initial position condition, it is assumed that the N-pole surface is located in the *βOγ* plane, and the S-pole surface is located in the *α* = −*h_m_* plane. The distance from the iron core surface to the N-pole surface of the magnet is *d*, and the coordinate origin is situated at the center of the magnet’s N-pole surface.

Within this coordinate system, the spatial geometric boundary conditions for the key components of the system are defined as follows:

Permanent magnet region: The axial span is *α* ∈ [−*h_m_*, 0], where the *α* = −*h_m_* plane represents the S-pole (negative magnetic charge distribution), and the *α* = 0 plane represents the N-pole (positive magnetic charge distribution). Here, *h_m_* denotes the height of the cylindrical permanent magnet and the iron core.

Coil and iron core motion region: In the initial stationary state, the gap between the end face of the iron core and the N-pole is *d*, and the axial coverage span of the coil and iron core is *α* ∈ [*d*, *h_m_* + *d*].

Based on the surface magnetic charge distribution theory, the permanent magnet is equivalent to the superposition of the magnetic fields generated by the polarized surface at *α* = 0 (with a surface magnetic charge density of +*σ_m_*) and the surface at *α* = −*h_m_* (with a surface magnetic charge density of −*σ_m_*), where *σ_m_* = *B_r_*/*μ*_0_. In the polar coordinate system (*r*,*θ*), the surfaces of the two poles are divided into infinitesimal integral elements. The conversion relationship between the polar coordinates and the spatial transverse coordinates is *β* = *r*cos*θ* and *γ* = *r*sin*θ*.

The expressions for the magnetic flux density along the *α*, *β*, and *γ* directions at any arbitrary point P(*α*,*β*,*γ*) in the space outside the permanent magnet are:

(20) $$B_{\alpha }(\alpha ,\beta ,\gamma )=\frac{B_{r}}{4\pi }\int_{0}^{R_{m}}r^{\prime }dr^{\prime }\int_{0}^{2\pi }\left[\frac{\alpha }{{\left[\alpha^{2}+\Delta {r^{\prime }}^{2}\right]}^{3/2}}-\frac{\alpha +h_{m}}{{\left[{\left(\alpha +h_{m}\right)}^{2}+\Delta {r^{\prime }}^{2}\right]}^{3/2}}\right]d\theta^{\prime }$$

(21) $$B_{\beta }(\alpha ,\beta ,\gamma )=\frac{B_{r}}{4\pi }\int_{0}^{R_{m}}r^{\prime }dr^{\prime }\int_{0}^{2\pi }\left[\frac{\beta -r^{\prime }\cos\theta^{\prime }}{{\left[\alpha^{2}+\Delta {r^{\prime }}^{2}\right]}^{3/2}}-\frac{\beta -r^{\prime }\cos\theta^{\prime }}{{\left[{\left(\alpha +h_{m}\right)}^{2}+\Delta {r^{\prime }}^{2}\right]}^{3/2}}\right]d\theta^{\prime }$$

(22) $$B_{\gamma }(\alpha ,\beta ,\gamma )=\frac{B_{r}}{4\pi }\int_{0}^{R_{m}}r^{\prime }dr^{\prime }\int_{0}^{2\pi }\left[\frac{\gamma -r^{\prime }\sin\theta^{\prime }}{{\left[\alpha^{2}+\Delta {r^{\prime }}^{2}\right]}^{3/2}}-\frac{\gamma -r^{\prime }\sin\theta^{\prime }}{{\left[{\left(\alpha +h_{m}\right)}^{2}+\Delta {r^{\prime }}^{2}\right]}^{3/2}}\right]d\theta^{\prime }$$

In the integral expression, the first term represents the contribution of the positive magnetic charge at the N-pole, and the second term represents the contribution of the negative magnetic charge at the S-pole. Δ*r* is the distance between point *P′* on the magnetic pole surface and the projection of a spatial point *P* onto the *βOγ* plane, which is expressed as:

(23) $$\Delta r^{2}={\left(\beta -r^{\prime }\cos\theta^{\prime }\right)}^{2}+{\left(\gamma -r^{\prime }\sin\theta^{\prime }\right)}^{2}$$

When the vibration of the two cantilever beams causes the coil and the high-permeability iron core to produce an instantaneous displacement *γ_c_*(*t*) along the *γ*-axis relative to the permanent magnet, the internal magnetic flux distribution is locally amplified due to the polarization characteristics of the iron core (flux concentration effect). The equivalent relative permeability of the iron core, *μ_reff_*, is introduced to correct for the influence of the demagnetization factor. Consequently, the total magnetic flux linkage *Ψ_m_*(*γ_c_*) passing through the *N*-turn coil, which is formed by the magnetic field of the iron core, is equivalent to the spatial average integral of the axial magnetic field inside the iron core over the effective volume of the coil:

(24) $$\Psi_{m}(\gamma_{c})=\frac{N}{h_{c}}\int_{d}^{d+h_{c}}d\alpha \iint_{S_{core}}\mu_{reff}\cdot B_{\alpha }(\alpha ,\beta ,\gamma )d\beta d\gamma$$

The expression for the effective relative permeability *μ_reff_* is given as follows:

(25) $$\mu_{reff}=\frac{\mu_{r}}{1+N_{d}(\mu_{r}-1)}$$

where *μ_r_* is the intrinsic relative permeability of the core material, and *N_d_* is the axial demagnetization factor of the finite cylindrical core. The demagnetization factor *N_d_* is a geometry-dependent quantity rather than a material constant, and is mainly determined by the aspect ratio of the cylindrical core, i.e.,

(26) $$p=\frac{h_{d}}{2R_{d}}$$

where *h_d_* and *R_d_* denote the height and radius of the iron core, respectively. A larger aspect ratio corresponds to a more slender core and a smaller demagnetization factor, whereas a smaller aspect ratio results in a stronger demagnetization effect and thus a larger *N_d_*.

After the iron core is magnetized within a non-uniform spatial magnetic field, the additional magnetic field energy stored by the system can be calculated through a volume integral:

(27) $$W_{m}(\gamma_{c})=\frac{\mu_{reff}-1}{2\mu_{0}}\int_{d}^{d+h_{m}}d\alpha \iint_{S_{core}}\left(B_{\alpha }^{2}+B_{\beta }^{2}+B_{\gamma }^{2}\right)d\beta d\gamma$$

Considering the self-inductance effect of the coil, the total magnetic flux linkage passing through the coil is expressed as:

(28) $$\Psi_{M}=\Psi_{m}+\Psi_{I}=\frac{N}{h_{c}}\int_{d}^{d+h_{c}}d\alpha \iint_{S_{core}}\mu_{reff}\cdot B_{\alpha }(\alpha ,\beta ,\gamma )d\beta d\gamma +L_{c}\cdot I$$

where *I* is the induced current generated by the coil, *L_c_* is the coil inductance, and *γ_c_* = *z*_2_ − *z*_1_ = −Δ*z*. The magnetic co-energy of the system *W_M_* is expressed as:

(29) $$W_{M}\left(\Delta z,I\right)=W_{m}\left(\Delta z\right)+I\cdot \Psi_{m}\left(\Delta z\right)+\frac{1}{2}L_{c}\cdot I^{2}$$

Furthermore, the nonlinear electromagnetic force *F_e_*(Δ*z*) exerted on the iron core along the vibration direction (*Z*-axis) can be obtained as:

(30) $$F_{e}(\Delta z)=\frac{\partial W_{M}(\Delta z,I)}{\partial \Delta z}=\frac{\partial W_{m}(\Delta z,I)}{\partial \Delta z}+I\cdot \frac{\partial \Psi_{m}\left(\Delta z\right)}{\partial \Delta z}$$

In the equation, the first term represents the nonlinear magnetic attractive force, namely the nonlinear static magnetic stiffness force *F_kz_*, while the second term represents the electromagnetic damping force *F_cz_* involving the coil current. When the coil moves relative to the magnet, an induced current *I* is generated, which produces a force opposing the motion, thereby realizing the conversion of mechanical energy into electrical energy.

Physically, *F_kz_* manifests as a nonlinear restoring force that resists the deviation of the cantilever beam from its equilibrium position. Its magnitude not only depends on the volumes of the permanent magnet and the iron core as well as the initial gap *d*, but also varies with *γ_c_* (i.e., Δ*z*), exhibiting the characteristics of a nonlinear elastic force. It is a key factor that alters the stiffness of the dual-cantilever system, thereby inducing multiple vibration modes. Expanding *F_kz_*(Δ*z*) using a Taylor series at the static equilibrium position (Δ*z* = 0) yields:

(31) $$F_{kz}(\Delta z)\approx {\left.\frac{\partial F_{kz}}{\partial (\Delta z)}\right|}_{\Delta z=0}\Delta z+\frac{1}{3!}{\left.\frac{\partial^{3}F_{kz}}{\partial {\left(\Delta z\right)}^{3}}\right|}_{\Delta z=0}{\left(\Delta z\right)}^{3}+\frac{1}{5!}{\left.\frac{\partial^{5}F_{kz}}{\partial {\left(\Delta z\right)}^{5}}\right|}_{\Delta z=0}+\cdot \cdot \cdot$$

The coefficient of the first-order term corresponds to the equivalent linear magnetic stiffness *K_m_*.

For *F_cz_*, substituting Equation (24) into Equation (30) yields the following expression:

(32) $$F_{cz}(\Delta z)=I\cdot \frac{\partial \Psi_{m}(\Delta z)}{\partial \Delta z}=I\cdot \frac{N}{h_{c}}\int_{d}^{d+h_{c}}d\alpha \iint_{S_{\mathrm{core}}}\mu_{\mathrm{reff}}\cdot \frac{\partial B_{\alpha }(\alpha ,\beta ,\Delta z)}{\partial \Delta z}d\beta d\gamma$$

When relative motion occurs between the iron core and the permanent magnet, the induced electromotive force (EMF) generated in the coil is:

(33) $$\varepsilon =\frac{d\Psi_{m}}{dt}=\frac{\partial \Psi_{m}(\Delta z)}{\partial \Delta z}\Delta \dot{z}$$

The circuit model for the coil and the external load resistance *R_l_* can be expressed as follows:

(34) $$(R_{c}+R_{l})I+L_{c}\dot{I}-\frac{\partial \Psi_{m}(\Delta z)}{\partial \Delta z}\Delta \dot{z}=0$$

where *R_c_* is the internal resistance of the coil, *L_c_* is the coil inductance, and *R_l_* is the external load resistance. During low-frequency vibration, the coil reactance is typically much smaller than the combined internal resistance and external load. In this case, the current within the coil can be approximately expressed as:

(35) $$I\approx \frac{\partial \Psi_{m}(\Delta z)/\partial \Delta z}{\left(R_{c}+R_{l}\right)}\Delta \dot{z}$$

By substituting Equation (35) into Equation (32), *F_cz_* can be approximately expressed as:

(36) $$F_{cz}(\Delta z)\approx \frac{{\left(\frac{\partial \Psi_{m}(\Delta z)}{\partial \Delta z}\right)}^{2}}{\left(R_{c}+R_{l}\right)}\Delta \dot{z}$$

Equation (36) indicates that *F_cz_* consists of a variable nonlinear coefficient multiplied by a velocity component, thereby exhibiting the characteristics of a nonlinear damping force. Based on the analyses of *F_kz_* and *F_cz_*, their respective physical properties—acting as an elastic force and a damping force—are verified. Consequently, the nonlinear force *F_ez_* generated during the electromagnetic induction process is modeled as an equivalent nonlinear spring-damper. From Equations (32) and (34), the expression for the electromagnetic electromechanical coupling coefficient *θ_m_* can be obtained as:

(37) $$\theta_{m}(\Delta z)=\frac{\partial \Psi_{m}\left(\Delta z\right)}{\partial \Delta z}=\frac{N}{h_{c}}\int_{d}^{d+h_{c}}d\alpha \iint_{S_{\mathrm{core}}}\mu_{\mathrm{reff}}\cdot \frac{\partial B_{\alpha }(\alpha ,\beta ,\Delta z)}{\partial \Delta z}d\beta d\gamma$$

The electromagnetic electromechanical coupling coefficient of the cantilever varies with Δ*z*, where *S_core_* represents the cross-sectional area of the cylindrical iron core. Finally, the electromechanical coupling model of the entire device is obtained as follows:

(38) $$\left\{\begin{array}{l} M_{1}{\ddot{z}}_{1}(t)+c_{1}{\dot{z}}_{1}(t)+K_{1}z_{1}(t)-\theta_{1}v_{1}(t)+F_{kz}(\Delta z)+\theta_{m}I=-M_{1}{\ddot{z}}_{b}(t)\\ C_{p1}{\dot{v}}_{1}(t)+\frac{v_{1}(t)}{R_{p1}}+\theta_{1}{\dot{z}}_{1}(t)=0\\ M_{2}{\ddot{z}}_{2}(t)+c_{2}{\dot{z}}_{2}(t)+K_{2}z_{2}(t)-\theta_{2}v_{2}(t)-F_{kz}(\Delta z)-\theta_{m}I=-M_{2}{\ddot{z}}_{b}(t)\\ C_{p2}{\dot{v}}_{2}(t)+\frac{v_{2}(t)}{R_{p2}}+\theta_{2}{\dot{z}}_{2}(t)=0\\ (R_{c}+R_{l})I+L_{c}\dot{I}-\theta_{m}\Delta \dot{z}=0 \end{array}\right.$$

### 2.3. Preliminary Determination of Dimensions and Modal Analysis

**Figure 3:**
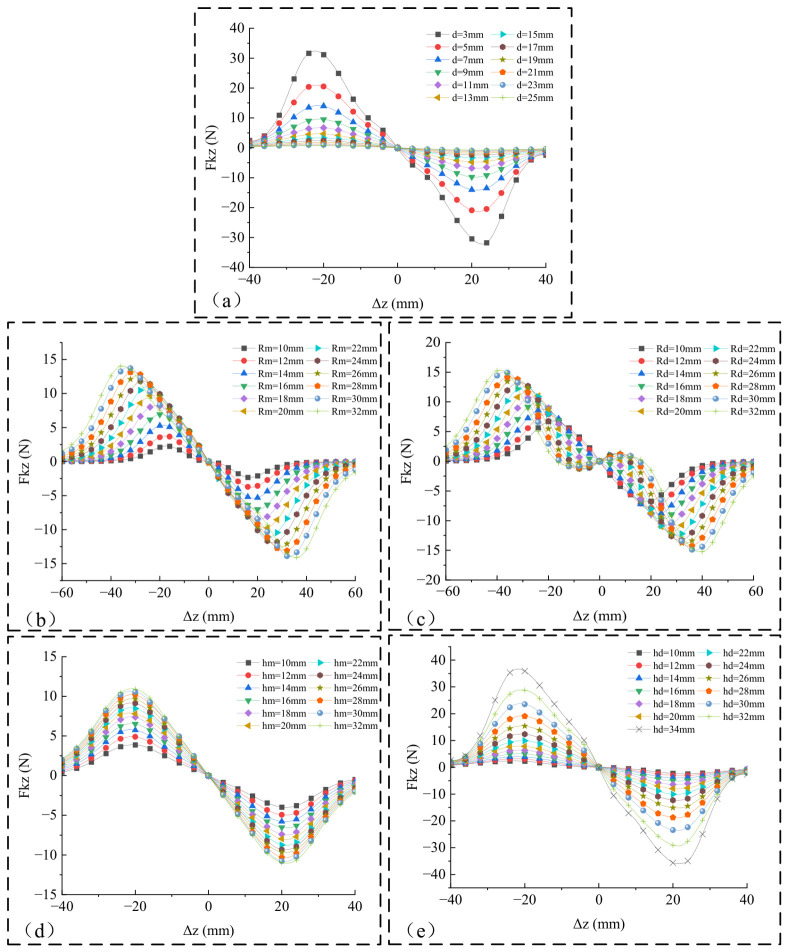
Variations in *F_kz_* with respect to Δ*z* under different parameters *d*, *R_m_*, *R_d_*, *h_m_*, and *h_d_*: (**a**) *F_kz_* under varying *d*; (**b**) *F_kz_* under varying *R_m_*; (**c**) *F_kz_* under varying *R_d_*; (**d**) *F_kz_* under varying *h_m_*; (**e**) *F_kz_* under varying *h_d_*.

The dimensions in Table 1 were obtained through preliminary simulation calculations and analysis. Initially, static simulations and analyses were conducted on the dimensions of the magnet and the iron-core coil. As shown in Figure 3, the nonlinear stiffness force *F_kz_* along the *Z*-axis is illustrated under various magnet and iron core dimensions. Specifically, the initial gap *d* plays a dominant role in determining *F_kz_*. As *d* increases, both the peak value of *F_kz_* and its slope near Δ*z* = 0 decay rapidly. When *d* exceeds 13 mm, the peak value of *F_kz_* approaches zero. To maximize the electromechanical coupling effect while maintaining a safety margin that prevents physical collisions of the cantilever beam, *d* should be kept as small as possible; therefore, *d* should be less than or equal to 13 mm.

**Figure 4:**
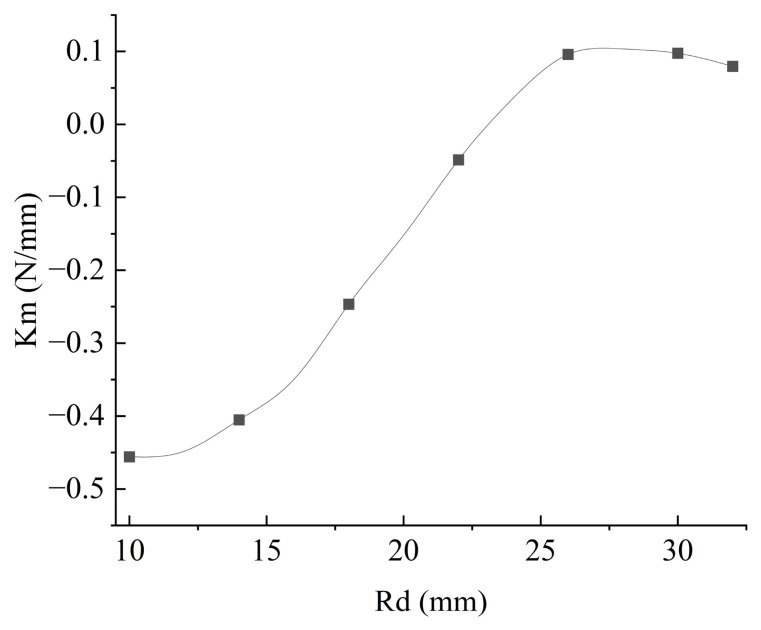
Variation in the equivalent stiffness coefficient *K_m_* with *R_d_*.

Meanwhile, the iron core dimensions *R_d_* and *h_d_* govern the volumetric effect of local magnetic flux concentration: increasing the height *h_d_* can steadily enhance the magnetic force amplitude. Although increasing the radius *R_d_* elevates the peak value, when *R_d_* is increased to 18 mm, it causes a distortion in *F_kz_*(Δ*z*). This further exacerbates the nonlinearity of *F_kz_*, complicating the motion process of the device and making it difficult to regulate the vibration response of the cantilever beam through parameter control. Furthermore, when *R_d_* > 25 mm, the equivalent stiffness coefficient *K_m_* of the first-order term—obtained via the Taylor expansion of *F_kz_* near Δ*z* = 0—exhibits a decreasing trend, as shown in Figure 4. This weakens the influence of *F_k__z_* on altering the cantilever beam’s stiffness, consequently making it difficult to shift the vibration modes of the cantilever beam.

**Figure 5:**
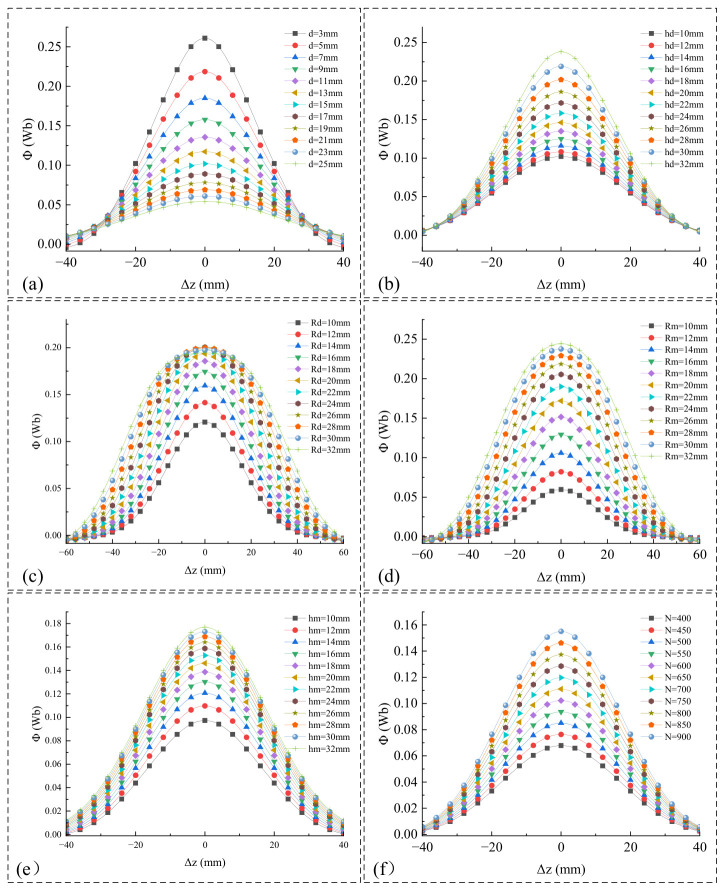
Variations in *Φ* with respect to Δ*z* under different parameters *d*, *h_d_*, *R_d_*, *R_m_*, *h_m_*, and *N*: (**a**) *Φ* under varying *d*; (**b**) *Φ* under varying *h_d_*; (**c**) *Φ* under varying *R_d_*; (**d**) *Φ* under varying *R_m_*; (**e**) *Φ* under varying *h_m_*; (**f**) *Φ* under varying *N*.

The total magnetic flux of the entire N-turn coil, *Φ*, is influenced not only by the geometric parameters of the magnet and the iron core but also by the coil parameters. To simplify the analysis process while considering practical conditions, it is assumed that the inner radius of the coil is equal to the radius of the iron core, and the height of the coil is identical to that of the iron core. COMSOL 6.2 was used to simulate the variations in *Φ* under different parameters *d*, *h_d_*, *R_d_*, *R_m_*, *h_m_*, and *N*, yielding the results shown in Figure 5 below:

The initial gap *d* most significantly affects the magnetic coupling strength, with *Φ* decaying as *d* increases. Therefore, *d* should be minimized while maintaining a safe margin to prevent physical collisions. The number of coil turns *N* exhibits a strictly linear and proportional relationship with *Φ*. Increasing the heights of the magnet *h_m_* and the iron core *h_d_* enhances *Φ*, but both exhibit significant saturation. Excessive increases in height yield diminishing returns and introduce excessive tip mass, which inevitably lowers the fundamental frequency of the cantilever beam. Enlarging the magnet radius *R_m_* effectively broadens the strong magnetic field region, significantly increasing *Φ*.

The design of the core radius *R_d_* is particularly critical. Based on the physical assembly assumption that the coil’s inner radius and height are identical to those of the core, increasing *R_d_* expands the coil’s outer radius without reducing *N* However, when *R_d_* > 18 mm, the growth of *Φ* plateaus or even decreases, indicating that *R_d_* should not be overly large.

Synthesizing the above trends, the parameter optimization strategy is proposed as follows: maximize *N* within the structural constraints of the sleeve to proportionally boost the induced electromotive force; and optimally match *R_d_* with *R_m_* such that *R_d_* is maximized while remaining smaller than *R_m_*. The final optimized dimensions of the iron-core coil and magnet are summarized in Table 1.

Once the parameters of the magnet and iron-core coil (such as *d*, *N*, *h_d_*, *R_d_*, *R_m_*, and *h_m_*) are fixed, the vibration modes of the device are governed by the cantilever length *L*, the thickness of cantilever *i* (*th_i_*), and the thickness difference between the two beams.

**Figure 6:**
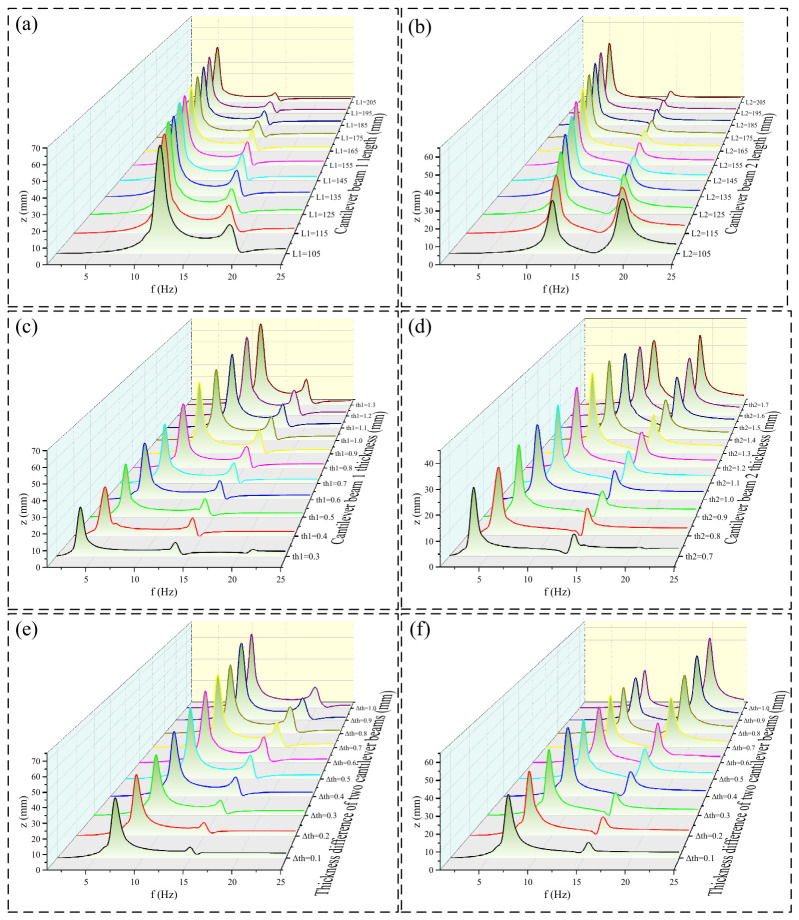
Frequency domain response of the vibration displacement at the end of cantilever beams under different cantilever beam parameters; (**a**) End displacement of cantilever beam 1 under different lengths, (**b**) End displacement of cantilever beam 2 under different lengths, (**c**) End displacement of cantilever beam 1 under different thicknesses, (**d**) End displacement of cantilever beam 2 under different thicknesses, (**e**) End displacement of cantilever beam 1 under different thickness differences, (**f**) End displacement of cantilever beam 2 under different thickness differences.

With the magnet-, core-, and coil-related parameters, including *d*, *N*, *h_d_*, *R_d_*, *R_m_*, and *h_m_*, kept constant, the vibration modes of the device are mainly affected by the cantilever length *L*, the thickness *th_i_* of cantilever *i*, and the thickness difference between the two cantilevers. Simulations of the device response under a constant excitation amplitude were carried out for different cantilever lengths, thicknesses, and thickness differences, as shown in Figure 6.

Under a sinusoidal excitation with an amplitude of 2 mm, the resonant frequencies of both cantilevers increase as the cantilever length decreases or as the thickness and thickness difference increase, and the two resonance peaks gradually approach each other. As shown in Figure 6a,b, when *L* decreases from 205 mm to 105 mm, the first resonant frequencies of the two cantilevers both increase from approximately 5.5 Hz to approximately 12 Hz, while the second resonant frequencies increase from approximately 14 Hz to approximately 19 Hz. As the cantilever length decreases, the steady-state average amplitude of cantilever 1 at the first resonant frequency increases from approximately 46 mm to approximately 64 mm, while that of cantilever 2 at the second resonant frequency increases from approximately 8 mm to approximately 30 mm. When L is reduced to 135 mm, the steady-state average amplitude of cantilever 1 at the second resonant frequency increases from approximately 7 mm to approximately 18 mm, after which it shows almost no further increase with decreasing cantilever length and even exhibits a slight downward trend. Similarly, the steady-state average amplitude of cantilever 2 at the first resonant frequency increases to approximately 40 mm, after which it remains nearly unchanged and also tends to decrease as the cantilever length continues to decrease.

As shown in Figure 6c,d, when $th_{1}$ increases from 0.3 mm to 1.3 mm and $th_{2}$ correspondingly increases from 0.7 mm to 1.7 mm, the first resonant frequencies of both cantilevers increase from approximately 3.5 Hz to approximately 12 Hz, while the second resonant frequencies increase from approximately 13.5 Hz to approximately 18.5 Hz. With increasing cantilever thickness, the tip amplitude of cantilever 1 at the first resonant frequency increases from approximately 30 mm to approximately 64 mm, while the steady-state average amplitude of cantilever 2 at the second resonant frequency increases from approximately 12 mm to approximately 37 mm. When $th_{1}$ reaches 1.0 mm, the steady-state average amplitude of cantilever 1 at the second resonant frequency increases from approximately 4.5 mm to approximately 18 mm, after which it shows little further improvement with increasing cantilever thickness and even tends to decrease slightly. When $th_{2}$ reaches 1.5 mm (corresponding to a thickness of 1.0 mm for cantilever 1), the steady-state average amplitude of cantilever 2 at the first resonant frequency increases to approximately 40 mm, after which it also remains nearly unchanged and shows a slight decreasing trend as the thickness continues to increase.

As shown in Figure 6e,f, when Δth increases from 0.1 mm to 1 mm, the first resonant frequencies of the two cantilevers increase from approximately 7 Hz to approximately 10.5 Hz, while the second resonant frequencies increase from approximately 15 Hz to approximately 20 Hz. As Δ*th* increases, the steady-state average amplitude of cantilever 1 at the first resonant frequency increases from approximately 38 mm to approximately 66 mm, while the steady-state average amplitude of cantilever 2 at the second resonant frequency increases from approximately 8 mm to approximately 30 mm. However, when Δ*th* reaches 0.5 mm, the steady-state average amplitude of cantilever 1 at the second resonant frequency increases from approximately 7 mm to approximately 18 mm, after which it shows almost no further increase with increasing thickness difference and even exhibits a slight downward trend. Likewise, the steady-state average amplitude of cantilever 2 at the first resonant frequency increases to approximately 40 mm, after which it remains nearly unchanged and also tends to decrease slightly as the thickness difference continues to increase.

Therefore, a cantilever length of 135 mm was preliminarily selected, while the thicknesses of cantilever 1 and cantilever 2 were set to 1.0 mm and 1.4 mm, respectively, so that both cantilevers exhibited relatively distinct dual-resonance peaks.

The vibration modes of the dual-cantilever system can be preliminarily identified through analytical modeling and simulation. To extract the system’s inherent vibration properties (i.e., characteristic modes), an undamped free vibration state is assumed. The nonlinear magnetic restoring force *F_kz_* is approximated using a first-order Taylor expansion around the static equilibrium position, yielding an equivalent linear coupling spring stiffness *K_m_*. By substituting *F_kz_* ≈ *K_m_*Δ*z* into the mechanical governing equations of the cantilevers and neglecting both damping and electromechanical coupling terms, the principal vibration equation of the system simplifies to the matrix form below:

(39) $$\left[\begin{array}{cc} M_{1} & 0\\ 0 & M_{2} \end{array}\right]\left\{\begin{array}{c} {\ddot{z}}_{1}\\ {\ddot{z}}_{2} \end{array}\right\}+\left[\begin{array}{cc} K_{1}+K_{m} & -K_{m}\\ -K_{m} & K_{2}+k_{m} \end{array}\right]\left\{\begin{array}{c} z_{1}\\ z_{2} \end{array}\right\}=\left\{\begin{array}{c} 0\\ 0 \end{array}\right\}$$

Assuming a harmonic free vibration solution of the form *z_i_*(*t*) = *Z_i_e^jωt^*, where *Z_i_* is the amplitude and *ω_l_* is the natural angular frequency of the system, the eigenvalue equation can be derived as:

(40) $$\left(K_{1}+K_{m}-{\omega_{l}}^{2}M_{1}\right)\left(K_{2}+K_{m}-{\omega_{l}}^{2}M_{2}\right)-K_{m}^{2}=0$$

**Figure 7:**
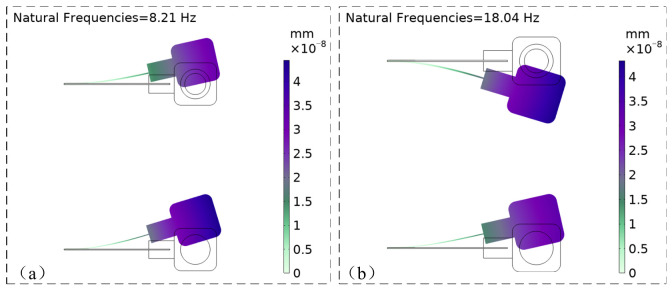
Two-dimensional (2D) plane representation of the cantilever vibration modes: (**a**) First resonant frequency point; (**b**) Second resonant frequency point.

It can be determined that the system possesses two resonant frequency points. Furthermore, using the parameters specified in Table 1, the vibration modes of the cantilever beams can be obtained through simulation as follows:

Figure 7 illustrates the vibration modes of the cantilever beams in a 2D plane (with Cantilever 2 at the top and Cantilever 1 at the bottom). At the first resonant frequency of 8.21 Hz, the two cantilever beams vibrate in phase. At the second resonant frequency of 18.04 Hz, the beams exhibit out-of-phase vibration. Notably, the mode shapes at both the first and second resonant frequencies are identical, characterized by maximum displacement at the free ends.

## 3. Results

Based on the aforementioned model, it can be readily derived that the structural parameters of the energy harvesting device, such as the initial gap *d*, exert a direct influence on its natural frequencies. By analyzing the effects of structural parameters like the gap *d* and the external load resistance *R_l_* on the performance of the energy harvester, the intricate details of its dynamic response and power generation capability are investigated in depth.

### 3.1. Open-Circuit Voltage of Piezoelectric Effect and Electromagnetic Induction Under Different Gaps d

**Figure 8:**
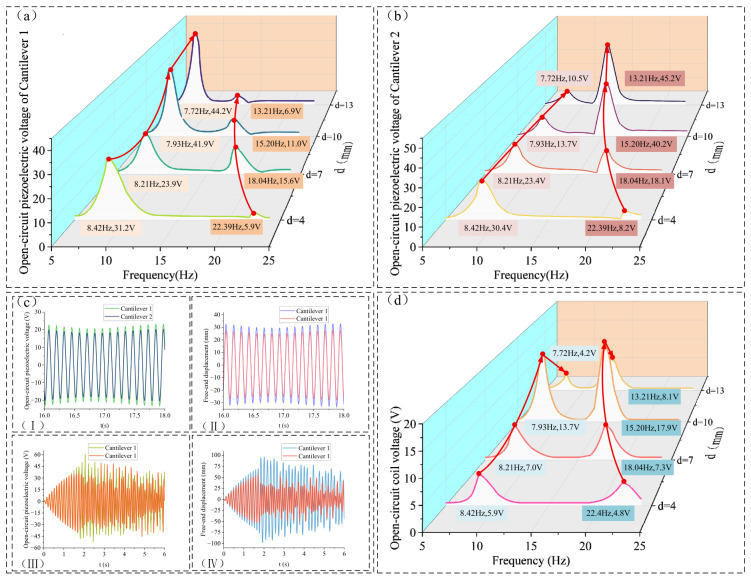
Open-circuit voltages of the piezoelectric films and coil versus frequency at different gap distances *d*: (**a**) open-circuit piezoelectric voltage of Cantilever 1; (**b**) open-circuit piezoelectric voltage of Cantilever 2; (**c**) piezoelectric output voltages and cantilever tip displacements under external excitation with amplitude of 2 mm and frequency of 8 Hz: (**I**) open-circuit piezoelectric voltage at *d* = 7 mm, (**II**) cantilever tip displacement at *d* = 7 mm, (**III**) open-circuit piezoelectric voltage at *d* = 10 mm, (**IV**) cantilever tip displacement at *d* = 10 mm; (**d**) open-circuit voltage of the coil.

It can be inferred from Figure 8b that *F_kz_* decreases continuously as the gap *d* increases. To investigate the impact of this variation on the output voltage response of the piezoelectric films and the coil, the effect of gap *d* on the output characteristics of the energy harvester was studied under the conditions of a 2 mm excitation amplitude and a frequency sweep from 5 to 10 Hz.

As shown in Figure 8a,b, when *d* = 4 mm, 7 mm, 10 mm, and 13 mm, the first resonance frequencies of the two cantilever beams are 8.42 Hz, 8.21 Hz, 7.93 Hz, and 7.72 Hz, respectively, while the second resonance frequencies are 22.39 Hz, 18.04 Hz, 15.20 Hz, and 13.21 Hz. As *d* increases, at the first resonance point, the piezoelectric open-circuit voltage of cantilever beam 1 first decreases and then increases, while the piezoelectric open-circuit voltage of cantilever beam 2 gradually decreases. At the second resonance point, the piezoelectric open-circuit voltage of cantilever beam 1 first increases and then decreases, while the piezoelectric open-circuit voltage of cantilever beam 2 gradually increases. When *d* = 4 mm, due to the large magnitude of *F_kz_*, the two cantilever beams approximately form a unified structure, and the bimodal response is less distinct; the piezoelectric open-circuit voltage resonance peak appears almost exclusively at 8.42 Hz, while the resonance peak at 22.39 Hz is inconspicuous.

When *d* increases from 7 mm to 10 mm, the piezoelectric open-circuit voltage of cantilever beam 1 at the first resonance frequency increases significantly from 23.9 V to 41.9 V, whereas the piezoelectric open-circuit voltage of cantilever beam 2 decreases from 23.4 V to 13.7 V. At the second resonance frequency, the piezoelectric open-circuit voltage of cantilever beam 2 increases sharply from 18.1 V to 40.2 V when *d* increases from 7 mm to 10 mm, while that of cantilever beam 1 decreases from 15.6 V to 11.0 V.

The primary reason for the above phenomena is the nonlinearity of *F_kz_*. As *d* increases from 7 mm to 10 mm, the peak value of *F_kz_* decreases, causing the tip displacement Δ*z* of the two cantilever beams to increase. As indicated in Figure 8b, when |Δ*z*| > 22 mm, the value of *F_kz_*(Δ*z*) no longer increases linearly but starts to decrease; at this point, the two cantilever beams approach a state with nearly no nonlinear interaction forces. For instance, at the first resonance frequency, the rapid reduction in the nonlinear force is insufficient to sustain the synchronization of cantilever beam 2 with the vibration of cantilever beam 1. Consequently, cantilever beam 2 decouples from the resonance state, leading to a sudden surge in the piezoelectric voltage of cantilever beam 1 and a reduction in that of cantilever beam 2.

This bimodal response behavior is illustrated in Figure 8c, which presents the piezoelectric open-circuit voltage and vibration displacement under an external excitation frequency of 8 Hz and an amplitude of 2 mm for *d* = 7 mm and *d* = 10 mm. Figure 8c(I,II) show the steady-state piezoelectric open-circuit voltage and tip displacement of the cantilever beams for *d* = 7 mm. To provide a more intuitive view of the decoupling process of cantilever beam 2, the results for *d* = 10 mm are output starting from 0 s. As shown in Figure 8c(III,IV), which depict the piezoelectric open-circuit voltage and tip displacement for *d* = 10 mm, when the excitation time approaches 2 s, cantilever beam 2 fails to follow the vibration displacement of cantilever beam 1 and decouples from the resonance state induced by the latter. The tip displacement and piezoelectric open-circuit voltage of cantilever beam 2 drop sharply from approximately 50 mm and 30 V to approximately 25 mm and 15 V, respectively.

The open-circuit voltage of the coil yields results similar to the piezoelectric voltage of the cantilever beams. When *d* ≤ 7 mm, the piezoelectric output results of the two cantilever beams are similar. At the first and second resonance points, the relative displacement of the cantilever beam tips increases with *d*, while the magnetic flux *Φ* of the coil decreases with *d*. Consequently, the induced electromotive force (EMF) of the coil remains relatively close for *d* = 4 mm and *d* = 7 mm.

**Figure 9:**
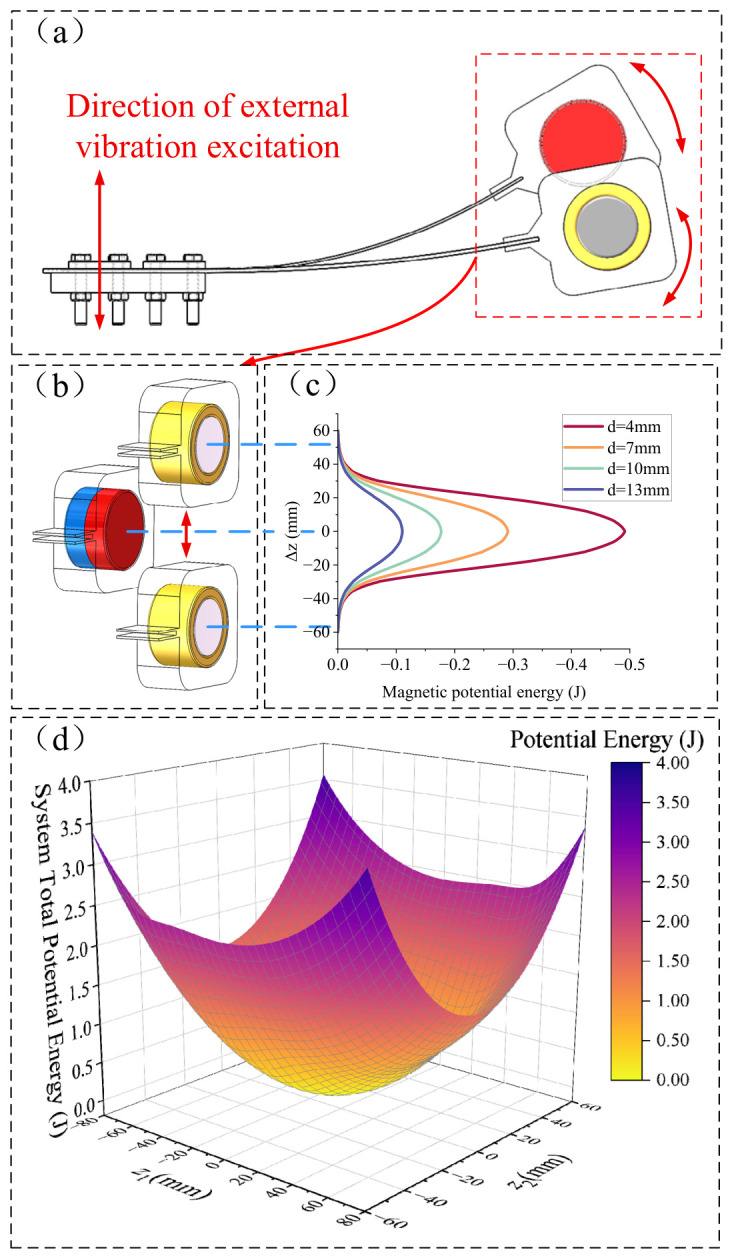
Correspondence between the permanent magnet potential wells and positions: (**a**) Device in a state of motion; (**b**) Relative displacement of the iron core with respect to the permanent magnet; (**c**) Magnetic potential energy corresponding to different displacements of the iron core relative to the permanent magnet; (**d**) Potential energy of the vibration energy harvesting device. The blue dotted line represents the coordinates corresponding to different relative displacements under different relative positions of the coil and the magnet, and the red double-headed arrow indicates the direction of motion.

During the transition from *d* = 7 mm to *d* = 10 mm, the sudden surge in the relative displacement between cantilever beam 1 and cantilever beam 2 causes the effect of the increased relative motion between the coil and the magnet to outweigh the reduction in magnetic flux, leading to a sharp increase in the induced EMF. The same conclusion regarding this bimodal system behavior can be drawn from the perspective of potential wells, as illustrated in Figure 9:

Figure 9a illustrates the relative displacement between the iron core and the permanent magnet during the vibration process. Figure 8b,c display the magnetic potential energy corresponding to different Z-direction displacements Δ*z* of the iron core relative to the permanent magnet (with the potential at infinity defined as the zero-potential surface). When Δ*z* = 0, the iron core is positioned at the potential well formed by the permanent magnet. Analogous to the analysis of potential wells formed by magnets under various lateral gaps in Zhao [32], this study conducts a simulation analysis of the moving potential well generated by the permanent magnet under different gaps *d* in the proposed bimodal method. When the gap $d$ is small, the iron core remains confined within the single potential well formed by the permanent magnet, thereby achieving coupling between the cantilever beams.

As *d* increases from 7 mm to 10 mm, the maximum potential well depth of the permanent magnet-iron core system decreases from 0.313 J to 0.175 J. In conjunction with the variations in tip displacement of the two cantilevers shown in Figure 8c(IV), the growth rate of the potential energy slows down when |Δ*z*| > 25 mm. As depicted in Figure 9c, when |Δ*z*| > 25 mm and the relative displacement increases further, the magnetic potential energy rapidly approaches zero, which is regarded as crossing the potential barrier. At this point, the interaction force *F_kz_* between the two cantilever beams loses its effectiveness, corresponding to the region in Figure 3 where *F_kz_* exceeds its nonlinear peak and enters a declining zone as Δ*z* increases. Consequently, the coupling effect between the two cantilevers weakens rapidly, leading to a significant reduction in the resonant displacement of cantilever 2 at the first resonance point and its eventual escape from the potential well. This also explains the sudden surge in Δ*z* and the induced electromotive force (EMF) of the coil. By utilizing the potential well formed by the permanent magnet, a coupling relationship between the two cantilever beams is established, enabling both beams to exhibit two vibration modes and thereby broadening the energy harvesting bandwidth of the piezoelectric films.

When *d* further increases to 13 mm, the maximum potential well depth decreases to 0.11 J, which further weakens the influence on the iron core and reduces the nonlinear interaction force between the two cantilevers. The piezoelectric output of cantilever 1 at the second resonance frequency and that of cantilever 2 at the first resonance frequency approach a non-resonant state. The impact of reduced magnetic flux outweighs the effect of the increased rate of change in magnetic flux caused by a larger Δ*z*, resulting in a significant decrease in the coil’s output voltage. The peak values of the induced EMF at the first and second resonance frequencies are only 4.2 V and 8.1 V, respectively.

Figure 9d illustrates the total potential energy of the device when *d* = 7 mm (with the static equilibrium position of the cantilever defined as the zero-potential plane), which comprises the elastic potential energy from the bending of the piezoelectric cantilevers and the magnetic potential energy between the magnet and the iron core. As observed in Figure 9d, the total potential energy forms a single potential well surface. This surface is not only determined by the vibration displacement of the cantilever on the magnet side but is also influenced by the displacement on the coil side. Moreover, the potential surface is not symmetric about *z*_1_ = 0 and *z*_2_ = 0; instead, it is influenced by the potential energy distribution of the permanent magnet and iron core. When |Δ*z*| exceeds 25 mm, the growth rate of the magnetic potential energy relative to Δ*z* decreases significantly, indicating that *F_kz_* has almost lost its effect. This leads to a certain offset in the potential surface; for instance, in Figure 9d, when *z*_2_ = 60 mm, the system’s potential energy at *z*_1_ = −80 mm is lower than that at *z*_1_ = 80 mm.

Based on the analysis of Figure 8 and Figure 9, when *d* = 10 mm, the peak value of *F_kz_* decreases, preventing cantilever beam 2 from continuously following the resonant displacement of cantilever beam 1. This leads to a sudden increase in the relative displacement between the tips of cantilever beams 1 and 2.

**Figure 10:**
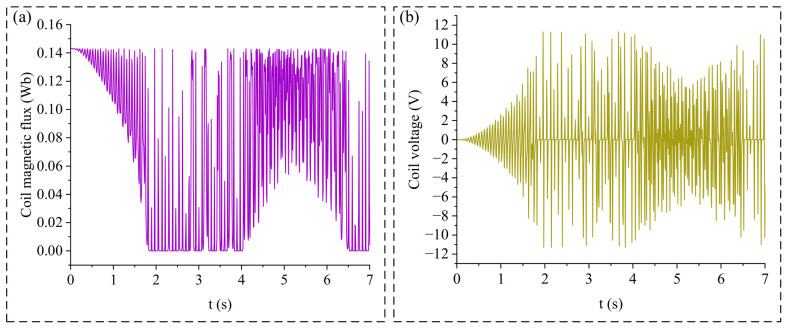
Variations in magnetic flux and coil voltage under external excitation with an amplitude of 2 mm, a frequency of 8 Hz, and a gap of *d* = 10 mm: (**a**) magnetic flux of the coil; (**b**) induced electromotive force of the coil.

Under this condition, the magnetic flux through the coil and the induced electromotive force (EMF) are shown in Figure 10, corresponding to the vibration displacement in Figure 8c(IV). At *t* = 1.8 s, cantilever beam 2 detaches from the resonant motion of cantilever beam 1, resulting in a large relative displacement. At this moment, the coil magnetic flux rapidly drops to 0 Wb and remains at this level for a short period. The relative displacement between the iron core coil and the magnet reaches approximately 55 mm.

As shown in Figure 5a, when *d* = 10 mm, the magnetic flux decreases to 0 Wb when Δz reaches about 40 mm. At this point, the iron core has moved out of the effective magnetic field region of the permanent magnet. As the relative displacement continues to increase, the magnetic flux remains at, 0 Wb and consequently, the induced EMF in the coil is also zero.

Furthermore, during the vibration process, cantilever beam 2 repeatedly transitions from following the resonant displacement of cantilever beam 1 to detaching from it (i.e., escaping from the potential well). At the moment of detachment, the magnetic flux in the coil undergoes a significant change. For example, at *t* = 2.2s in Figure 10a, the magnetic flux abruptly decreases from 0.142 Wb to 0 Wb, which exactly corresponds to the instant when cantilever beam 2 detaches from the resonant state of cantilever beam 1, resulting in a sharp increase in the induced EMF.

Compared with the magnet-array-based approaches reported in refs. [33,34], the mechanism for enhancing the rate of change in magnetic flux density (*dϕ/dt*) in this work is fundamentally different.

In refs. [33,34], abrupt changes in magnetic flux density are intentionally introduced through alternating-polarity magnet arrays. In such configurations, the magnetic flux undergoes rapid spatial transitions at the junctions between adjacent magnets, resulting in multiple high-frequency *dϕ/dt* peaks. Therefore, the enhancement of *dϕ/dt* primarily originates from spatial polarity reversals, where both the magnitude and abruptness of flux variation are governed by magnet arrangement and polarity distribution.

In contrast, the present work does not rely solely on spatial discontinuities of the magnetic field, but instead enhances *dϕ/dt* through a kinematic mechanism induced by nonlinear dynamic behavior. As shown in Figure 10a, when cantilever beam 2 detaches from the resonant motion (e.g., at *t* = 2.2 s), the magnetic flux through the coil abruptly decreases from 0.142 Wb to 0 Wb within a very short time interval, resulting in a significantly large transient *dϕ/dt*.

Therefore, compared with magnet-array-based designs, the proposed system exhibits distinct *dϕ/dt* characteristics: (1) a larger peak magnitude of *dϕ/dt* due to the instantaneous loss of magnetic coupling; (2) flux variation induced by abrupt relative displacement rather than spatial polarity reversal; (3) *dϕ/dt* peaks governed by dynamic instability (resonance detachment) instead of fixed spatial locations.

These results indicate that, while existing studies enhance *dϕ/dt* through magnetic field structural design, the present work achieves flux variation enhancement via nonlinear dynamic amplification, providing an alternative approach for improving electromagnetic energy harvesting performance.

### 3.2. Open-Circuit Voltage of Piezoelectric Effect and Electromagnetic Induction Under Different Amplitudes

**Figure 11:**
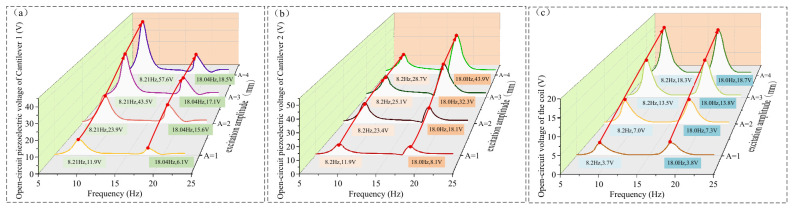
Open-circuit voltage outputs of the piezoelectric films and the coil under different external excitation amplitudes: (**a**) open-circuit voltage of cantilever beam 1; (**b**) open-circuit voltage of cantilever beam 2; (**c**) open-circuit voltage of the coil.

Based on the variation in piezoelectric voltage in Figure 8 and the potential well depth in Figure 9 as a function of *d*, it can be observed that when the iron core fails to escape from the potential well formed by the permanent magnet (e.g., when *d* = 7 mm and the external excitation amplitude is 2 mm), the piezoelectric voltage exhibits distinct bimodal vibration response characteristics. When the iron core escapes from the potential well, the bimodal characteristics of the piezoelectric voltage weaken, and a portion of the vibration energy is transferred from the piezoelectric output to the coil output. In addition to reducing the potential well depth by increasing *d*, increasing the external excitation amplitude similarly provides sufficient vibration energy for the iron core to escape from the potential well formed by the permanent magnet. As shown in Figure 11, the open-circuit voltage output results of the piezoelectric films and the coil under different external excitation amplitudes for *d* = 7 mm are as follows:

As can be observed from Figure 11, as the amplitude *A* increases, both the piezoelectric voltage and the coil voltage exhibit an upward trend. When *A* ≤ 2 mm, the vibration displacement of the cantilever beams is such that the iron core fails to escape from the potential well formed by the permanent magnet; consequently, the piezoelectric and coil voltages increase almost proportionally with the amplitude. However, when *A* increases from 2 mm to 3 mm, the piezoelectric voltage of cantilever beam 1 at the first resonance point increases significantly from 23.9 V to 43.5 V, while at the second resonance point, the voltage only increases from 15.6 V to 17.1 V.

The reason for this is that the increased amplitude enhances the energy obtained by the cantilever beams from the external environment. During the vibration process, the iron core can escape from the potential well formed by the permanent magnet, leading to a substantial improvement in the piezoelectric output of cantilever beam 1 at the first resonance point and cantilever beam 2 at the second resonance point, which further characterizes the bimodal response. Conversely, the effect of increasing amplitude on the piezoelectric voltage of cantilever beam 1 at the second resonance point is limited, which is also due to the constraint of *F_kz_*. As the amplitude increases, the relative displacement Δ*z* at the end of the cantilever beams increases. Once the vibration displacement at the beam end exceeds 22 mm, *F_kz_* decreases rapidly, making it impossible to maintain the state where cantilever beam 2 follows the vibration of cantilever beam 1. As a result, the influence of the increased amplitude on the piezoelectric voltage of cantilever beam 2 at the second resonance point is similarly limited.

When the amplitude *A* increases from 2 mm to 3 mm, the coil voltage shows a significant increase. Specifically, at the first and second resonance frequencies, the coil voltage increases from 7.0 V and 7.3 V to 13.5 V and 13.8 V, respectively. Unlike the piezoelectric output voltage, when *A* ≥ 3 mm, the output voltages of the coil at both resonance points continue to increase with the amplitude. Based on the variations in the piezoelectric and coil voltages with *d* and *A* in Figure 8 and Figure 11, the characteristics of the piezoelectric and electromagnetic outputs are as follows:

When the external excitation amplitude *A* and the gap *d* between the two cantilever beams are small, the iron core does not escape from the potential well formed by the permanent magnet. In this state, the coupling between the two cantilever beams is achieved through the potential well, enabling each cantilever beam to exhibit distinct bimodal vibration characteristics, which realizes the broadband energy harvesting performance of the piezoelectric effect.

Furthermore, under low external excitation amplitudes, the piezoelectric output of each cantilever beam presents a dominant main peak and a relatively lower secondary peak. Simultaneously, the coil’s output voltage improves significantly, showing two distinct peaks with similar values when *d* = 7 mm.

### 3.3. Optimal Load Resistance

**Figure 12:**
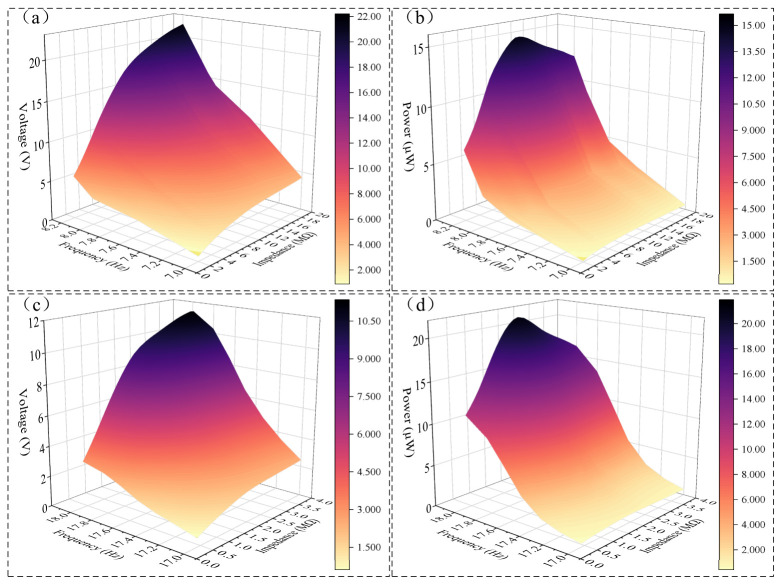
Output voltage and load power of cantilever beam 1 at the first and second resonant frequencies under various piezoelectric loads: (**a**) piezoelectric output voltage near the first resonance; (**b**) piezoelectric load power near the first resonance; (**c**) piezoelectric output voltage at the second resonance; (**d**) piezoelectric load power near the second resonance.

**Figure 13:**
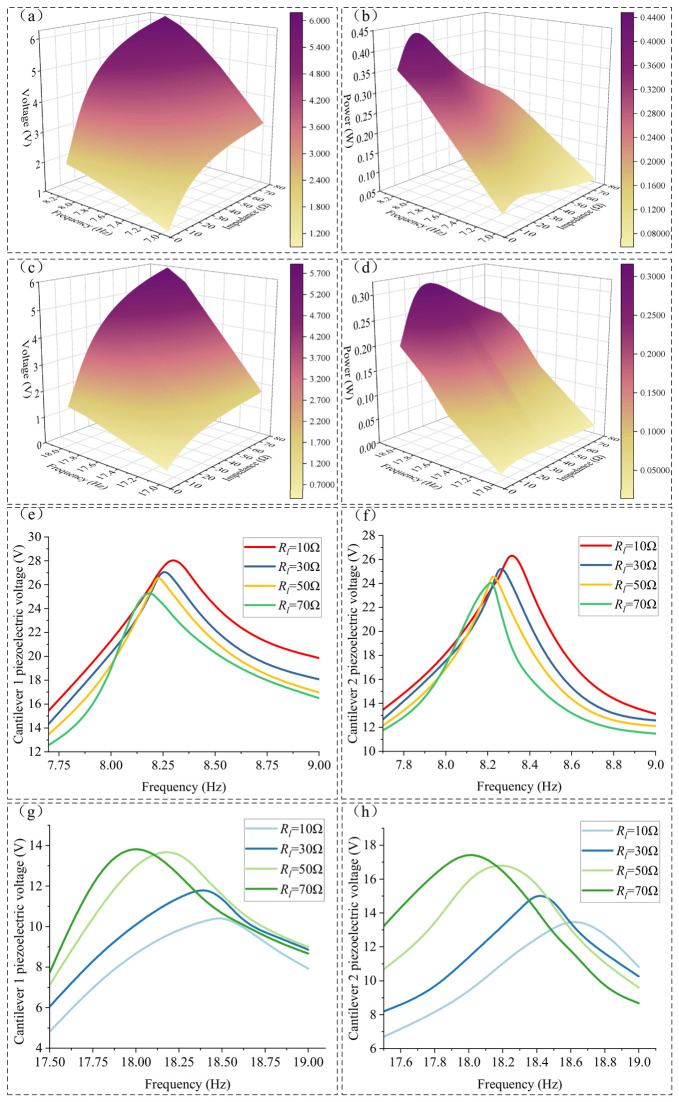
Coil output voltage and load power at the first and second resonant frequencies under various coil loads: (**a**) output voltage near the first resonance; (**b**) load power near the first resonance; (**c**) output voltage near the second resonance; (**d**) load power near the second resonance; (**e**) piezoelectric open-circuit voltage of cantilever beam 1 near the first resonance; (**f**) piezoelectric open-circuit voltage of cantilever beam 2 near the first resonance; (**g**) piezoelectric open-circuit voltage of cantilever beam 1 near the second resonance; (**h**) piezoelectric open-circuit voltage of cantilever beam 2 near the second resonance.

The output power of the piezoelectric film is significantly influenced by the external resistance. To evaluate the output power of the bimodal vibration energy harvesting device, the results for the piezoelectric film with different external resistances *R_y_* and the coil with different external resistances *R_l_* are shown in Figure 12 and Figure 13, respectively, under the conditions of a 2 mm external excitation amplitude and d = 7 mm.

Since the matched impedance of the piezoelectric film is significantly affected by the vibration frequency, and the resonance frequencies of cantilever beam 1 and cantilever beam 2 are identical when *d* = 7 mm, the output voltage and output power of the piezoelectric film on cantilever beam 1 under different external load resistances at the first and second resonance points were calculated and analyzed. The results are shown in Figure 12.

Near the first resonance point (8.21 Hz), the output voltage of the piezoelectric film increases as *R_y_* increases. When *R_y_* > 10 MΩ, the growth rate of the piezoelectric voltage decreases and gradually approaches the open-circuit voltage. At *R_y_* = 10 MΩ, the peak resonance output voltage of the piezoelectric film is 17.2 V. As shown in Figure 12b, the piezoelectric film reaches its maximum output power of 14.8 μW when *R_y_* = 10 MΩ; however, when *R_y_* > 10 MΩ, the output power of the piezoelectric film gradually decreases as *R_y_* increases.

Near the second resonance frequency (18.04 Hz), the output voltage of the piezoelectric film increases as *R_y_* increases. When *R_y_* > 2.3 MΩ, the growth rate of the piezoelectric voltage decreases and gradually approaches the open-circuit voltage. At *R_y_* = 2.3 MΩ, the peak resonance output voltage of the piezoelectric film is 9.84 V. As shown in Figure 12d, the piezoelectric film reaches its maximum output power of 21.05 μW when *R_y_* = 2.3 MΩ; however, when *R_y_* > 2.3 MΩ, the output power of the piezoelectric film gradually decreases as *R_y_* increases.

Regarding the different external load resistances *R_l_* for the coil, it can be inferred from Equations (32) and (35) that changes in *R_l_* will lead to variations in the electromagnetic damping force *F_cz_*. This further alters the vibration displacement at the end of the cantilever beams and the relative displacement Δ*z* between the iron core and the coil, thereby influencing the amplitude of the induced electromotive force (EMF) and the output power of the coil.

The output voltage and power of the coil near the first and second resonance frequencies, as well as the variation in the piezoelectric film’s open-circuit voltage with *R_l_* for this bimodal device, are shown in Figure 13.

Near the first resonance point, when *R_l_* = 14 Ω, the peak output voltage of the coil is 3.5 V, and the output power reaches its maximum value of 0.4375 W. When *R_l_* > 14 Ω, the growth rate of the coil’s output voltage with increasing load gradually decreases, and the output power decreases.

Near the second resonance point, when *R_l_* = 26 Ω, the peak output voltage of the coil is 4.2 V, and the maximum output power is 0.339 W.

As illustrated in Figure 13e through Figure 13h, a decrease in *R_l_* leads to a gradual increase in *F_cz_*, which subsequently shifts the resonant frequencies of both cantilever beams higher. Specifically, when *R_l_* = 10 Ω, the first resonant frequency increases to 8.33 Hz (compared to 8.21 Hz at *R_l_* = ∞), and the second resonant frequency reaches 18.48 Hz (compared to 18.04 Hz at *R_l_* = ∞). At the first resonance point, the peak voltage of the piezoelectric film increases as *R_l_* decreases, whereas the opposite trend is observed at the second resonance point. Comparing these results with Figure 13, the effect of increasing *F_cz_* on the piezoelectric voltage mirrors that of decreasing *d*; both lead to the two cantilevers behaving more like a single integrated unit.

This phenomenon is attributed to the nature of the damping force and the specific vibration modes of the cantilevers. Analysis of Figure 13 reveals that at the first resonant frequency, the vibration displacements at the free ends of Cantilever 1 and Cantilever 2 are in phase. In this state, the damping force *F_cz_* causes the two beams to vibrate more as a unified body (or effectively enhances the coupling between them). Conversely, at the second resonant frequency, the displacements at the free ends are out of phase. Here, the damping effect of the coil force on the relative displacement between the two beams becomes particularly pronounced. As the damping force increases, the tip displacements of both cantilevers decrease, leading to a reduction in piezoelectric output voltage as the vibration energy is converted into electromagnetic output energy.

## 4. Experiment and Analysis

### 4.1. Experimental Platform and Instruments

**Figure 14:**
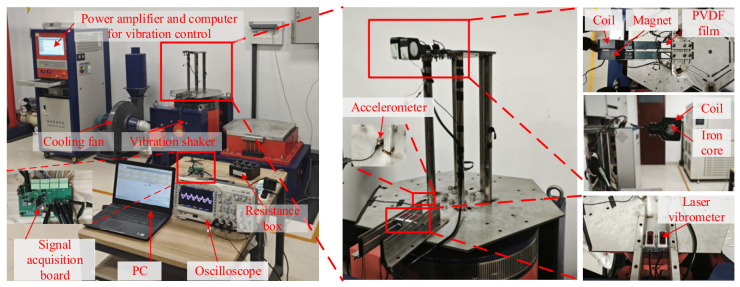
Vibration platform and test setup.

In addition to the simulation calculations, a prototype of the device was fabricated and experiments were conducted in this study, as shown in Figure 14. The experimental platform consists of a Jianqiao JQA-062-23 horizontal-vertical vibration table (comprising a computer, power amplifier, heat sink, and the vibration table itself), a RIGOL MSO4024 oscilloscope, a Jingjiake ZW-LV400T laser displacement sensor, a 0.1–9999.9 Ω adjustable resistance box, a 316 stainless steel connection bracket, a computer, and the vibration energy harvesting device.

The vibration platform is utilized to provide vertical sweeping vibration (in the *z*-direction) with an amplitude of 2 mm and a frequency range from 5 Hz to 20 Hz. The laser displacement sensor is used to record the *z*-direction displacement data at the end of the cantilever beam and transmit it to the computer, while the oscilloscope records the output voltage of the piezoelectric film at the root of the cantilever beam. Furthermore, to test the matching impedance of the PVDF film, several high-precision resistors (1 MΩ, 2 MΩ, 4 MΩ, 6 MΩ, 8 MΩ, 10 MΩ, 20 MΩ, and 200 MΩ) were prepared.

### 4.2. Experimental Results and Analysis

**Figure 15:**
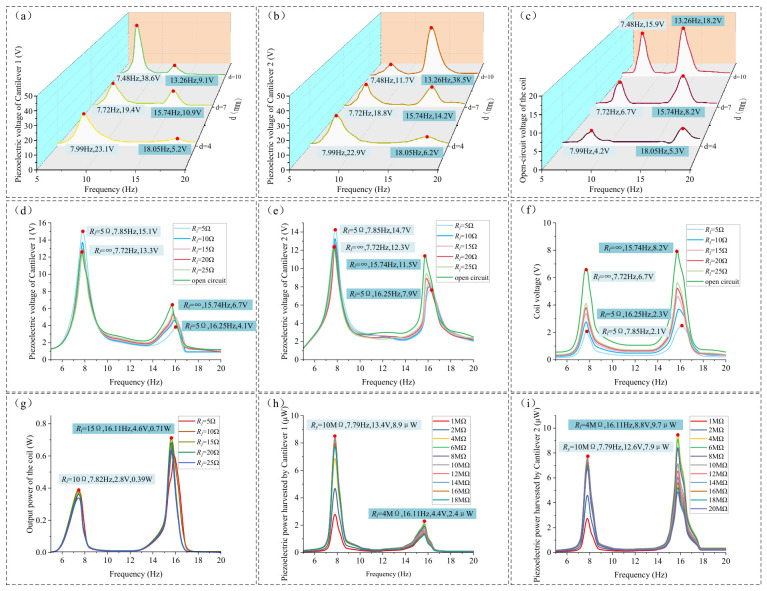
Experimental results: (**a**) Open-circuit voltage frequency sweep of cantilever 1 with varying gap distance d; (**b**) Open-circuit voltage frequency sweep of cantilever 2 with varying gap distance d; (**c**) Open-circuit voltage frequency sweep of the coil with varying gap distance d; (**d**) Cantilever 1 piezoelectric output voltage versus coil load; (**e**) Cantilever 2 piezoelectric output voltage versus coil load; (**f**) Coil output voltage versus coil load; (**g**) Coil output power versus coil load; (**h**) Cantilever 1 piezoelectric output power versus piezoelectric load; (**i**) Cantilever 2 piezoelectric output power versus piezoelectric load.

Under the conditions of a 2 mm amplitude and a 5–20 Hz frequency sweep, the open-circuit voltage results (with a 200 MΩ load) for the piezoelectric films and the coil at different gaps *d* were obtained, as shown in Figure 15a–c. (Constrained by the vibration shaker’s rated amplitude (not exceeding 2.5 mm) and maximum continuous operating duration (not exceeding 10 min), the frequency sweep tests were conducted only at an amplitude of 2 mm).

As illustrated in Figure 15a–c, the variation in the open-circuit voltage with *d* aligns closely with the simulation results. As *d* increases, the piezoelectric output of the cantilever beams follows a pattern of transitioning from a nearly single peak to distinct bimodal resonance peaks, and then back to a nearly single resonance peak, which is consistent with the simulation findings. With the increase in *d*, the first resonance frequency decreases from 7.99 Hz to 7.48 Hz, and the second resonance frequency decreases from 18.05 Hz to 13.26 Hz. Both the resonance frequencies and the piezoelectric open-circuit voltages are lower than the simulation results. This discrepancy may be attributed to the fact that the actual stiffness of the cantilever beams is lower than the ideal values set in the simulation. When *d* ≥ 7 mm, the peak voltages at various points are as indicated by the labels in the figures. Measurements showed that the actual thicknesses of cantilever 1 and cantilever 2 were 0.98 mm and 1.39 mm, respectively, which are slightly lower than the simulation values of 1.0 mm and 1.4 mm. In addition, the supplier indicated that the Young’s modulus of the cantilevers was approximately 200 GPa, which is also slightly lower than the simulation value of 206 GPa. Furthermore, the actual parameters of the magnet and iron core did not fully match the values used in the simulation. For example, the measured diameter of the permanent magnet was 33.8 mm, and the relative permeability of the iron core was also slightly lower than the simulated value, with the supplier indicating a value of approximately 3500.

After recalculating the first and second resonant frequencies using the actual parameters, the simulation results were found to be closer to the experimental measurements, with the errors remaining within an acceptable range, which to some extent validates the accuracy of the simulation model (see Table 2).

Figure 15d–g illustrate the effects of different external coil resistances on the piezoelectric film (at a 10 MΩ load) and the coil output voltage, as well as the output power of the coil. A larger external coil resistance results in a smaller *F_cz_*, which reduces the influence on the motion of the two cantilever beams and, consequently, minimizes the impact on the output voltage of the piezoelectric film.

As observed in Figure 15d,e, when *R_l_* decreases from ∞ to 5 Ω, the first resonance frequency increases from 7.72 Hz to 7.85 Hz. At this first resonance frequency, the piezoelectric output voltages of cantilever beam 1 and cantilever beam 2 increase from 13.3 V and 12.3 V to 15.1 V and 14.7 V, respectively. The second resonance frequency increases from 15.74 Hz to 16.25 Hz; at this frequency, the piezoelectric output voltages of cantilever beam 1 and cantilever beam 2 decrease from 6.7 V and 11.5 V to 4.1 V and 7.9 V, respectively.

The experimental results show that the trends in resonance frequency shifts and piezoelectric film output voltage variations caused by *F_cz_* are consistent with the simulation results, shifting the two cantilever beams toward a more unified structure in this bimodal system. Compared to the simulation, the degree of resonance frequency shift and piezoelectric output voltage change caused by the actual *F_cz_* is smaller than the results obtained in Figure 13.

According to Figure 15f,g, at the first resonance frequency, the matched impedance of the coil is 10 Ω, the load voltage is 2.8 V, and the output power is 0.39 W. At the second resonance frequency, the matched impedance of the coil is 15 Ω, the load voltage is 4.6 V, and the output power is 0.71 W.

The discrepancy observed above is attributed to the difference in the relative magnetic permeability of the iron core. According to the manufacturer, the actual relative permeability of the core is approximately 3500, whereas the default value for iron in the COMSOL simulation is 4000. This variance resulted in a deviation in reactance; specifically, the simulated reactance values at the first and second resonant frequencies were 3.34 Ω and 7.51 Ω, respectively, while the corresponding measured values were 2.49 Ω and 5.66 Ω. Furthermore, the coil turns and internal resistance differed slightly from the simulation settings. The measured coil consists of approximately 600 turns with an internal resistance of 10 Ω, compared to the simulation’s 700 turns and a calculated resistance of 13.3 Ω. These factors collectively explain why the experimentally determined matched impedance is lower than that obtained through simulation.

Figure 15h,i illustrate the output power of the piezoelectric film under the conditions of a 15 Ω coil load and various external loads *R_y_*. At the first resonance point, the matched impedance of the piezoelectric film is 10 MΩ. The output power and load voltage for cantilever beam 1 are 8.9 μW and 13.4 V, respectively, while for cantilever beam 2, the output power is 7.9 μW and the load voltage is 12.6 V.

At the second resonance frequency, the matched impedance of the piezoelectric film is 4 MΩ. For cantilever beam 1, the piezoelectric film output power is 2.4 μW with a load voltage of 4.4 V; for cantilever beam 2, the output power reaches 9.7 μW with a load voltage of 8.8 V. Due to the fact that the performance of the actual piezoelectric film does not reach the theoretical values used in the simulation, the resulting output power and voltage are lower than the simulation results for this bimodal energy harvesting device.

**Figure 16:**
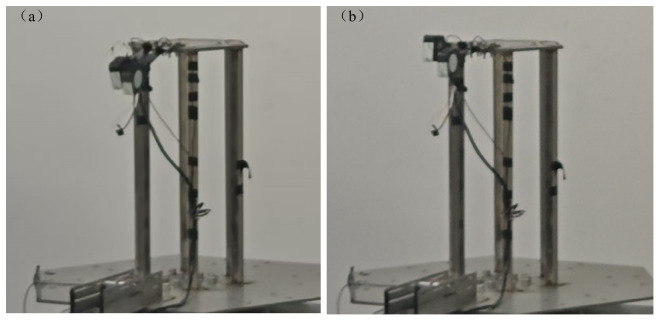
Vibration modes of the cantilever beams at different resonant frequencies: (**a**) In-phase vibration at the first resonant frequency; (**b**) Out-of-phase vibration at the second resonant frequency.

At the same time, the vibration behavior of the cantilever beams at both the first and second resonant frequencies was recorded, yielding the results shown in Figure 16 below:

It is observed that the beams exhibit in-phase vibration at the first resonant frequency, whereas the vibrations are out of phase (with the two cantilevers moving in opposite directions) at the second resonant frequency. In both cases, the mode shapes are characterized by maximum displacement at the free ends of the cantilevers, which is consistent with the analytical model and simulation results.

### 4.3. Comparative Analysis

**Figure 17:**
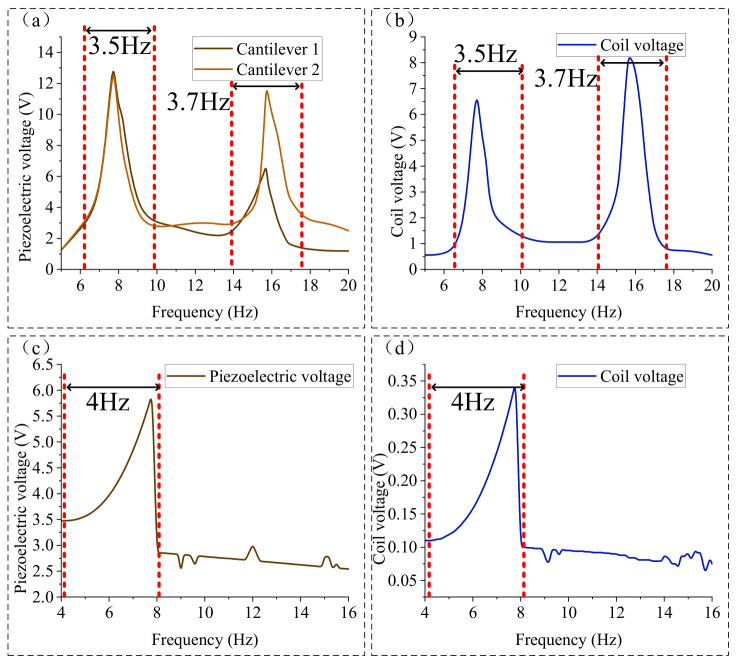
Comparison of broadband performance: (**a**) Voltage frequency response curves of the dual-mode device (10 MΩ load for piezoelectric film); (**b**) Voltage frequency response curves of the dual-mode device coil (1 kΩ load); (**c**) Voltage frequency response curves of the bistable device (1 MΩ load for piezoelectric film); (**d**) Voltage frequency response curves of the bistable device coil (1 kΩ load).

Synthesizing the experimental results above, it is observed that the proposed dual-mode piezo-electromagnetic coupled vibration energy harvester exhibits high output power across frequency ranges of 6.3–9.8 Hz and 14–17.7 Hz under an excitation amplitude of 2 mm. To further verify the broadband performance of the proposed device, its output voltage frequency response (at a piezoelectric load of 10 MΩ and a coil load of 1000 Ω) is compared with the single-mode bistable harvester reported in ref. [26]. The comparison is illustrated in Figure 17:

From the comparison results in Figure 17, it can be observed that the bistable vibration energy harvesting device primarily achieves a broadband effect by extending the bandwidth near the resonance frequency, with an effective working frequency band of approximately 4–8 Hz. In contrast, the piezoelectric–electromagnetic coupled bimodal vibration energy harvesting device proposed in this study achieves the broadband effect by generating multiple vibration modes.

The proposed device offers total effective working bandwidths of 6.3–9.8 Hz and 14–17.7 Hz, amounting to a combined 7.2 Hz (Two discontinuous working frequency bands totaling 7.2 Hz.), which represents a bandwidth improvement of approximately 80%. Furthermore, the piezoelectric and coil voltages near each resonance frequency exhibit higher amplitudes. Compared with the bistable energy harvester, the piezoelectric voltage at the first resonance frequency increased from 5.94 V to 13.3 V, an improvement of 123.91%.

At the second resonance frequency, the piezoelectric voltage increased from 2.78 V to 11.3 V (the peak voltage of cantilever beam 2), representing a significant improvement of 306.47%. The piezoelectric output voltage within the widened frequency band shows a substantial increase. Additionally, due to the presence of the iron core, the improvement in the coil voltage output is even more pronounced.

The output performance of the proposed device is compared with those of various harvesters reported in the literature, as summarized in Table 3. Notably, calculations indicate that the external excitation conditions for the proposed device at the first resonant frequency (frequency of 8 Hz and amplitude of 2 mm) are equivalent to an excitation of 0.5 g acceleration at 8 Hz.

The meanings of the symbols in Table 3 are as follows: a: Acceleration, f: Frequency (Hz), M: Material, S: Size (mm^3^), MI: Matching Impedance (Ω), N: Turns, D: Diameter (mm), R: Internal Resistance (Ω), H: Height (mm), WD: Wire Diameter (mm), MP: Magnetic Properties, ID: Inner Diameter (mm), OD: Outer Diameter (mm).

A structural comparison with other devices reveals that the electromagnetic induction performance of the proposed harvester is superior. This enhancement is attributed to the larger dimensions of the selected coil and permanent magnet, particularly the magnet diameter of 34 mm. Furthermore, the coil wire diameter is approximately three times thicker than those used in other devices, which significantly reduces the internal resistance and further boosts the output power.

## 5. Conclusions

### 5.1. Summary

This paper proposes a bimodal vibration energy harvesting method that utilizes the nonlinear force generated during the electromagnetic induction process to achieve piezoelectric–electromagnetic coupling. A parallel-arranged dual-cantilever beam structure was designed. Based on simulation calculations of the dynamic model and experimental validation of the device, the following primary conclusions are drawn:

1. The attraction force *F_kz_* between the permanent magnet and the iron core enables the dual-cantilever beams to exhibit bimodal resonance modes (7.72 Hz and 15.74 Hz) within the 5–20 Hz range. *F_kz_* decreases as the gap *d* increases, forming a dynamic potential well with an adjustable depth. At *d* = 4 mm, the large magnitude of *F_kz_* causes the two cantilever beams to vibrate synchronously as a unified structure. The simulation results show a distinct piezoelectric open-circuit voltage peak at 8.42 Hz, while the second resonance peak at 22.39 Hz is inconspicuous. At *d* = 7 mm, with the iron core located within the potential well, the piezoelectric open-circuit voltages of cantilever beam 1 and cantilever beam 2 at the first resonance point are 23.9 V and 23.4 V, respectively, showing a significant bimodal response. At *d* = 10 mm, the potential well becomes shallower, and the iron core escapes the well, leading to the decoupling of the two cantilever beams. At the first resonance point, the voltage of cantilever beam 1 rises to 41.9 V while cantilever beam 2 drops to 13.7 V. At the second resonance point, the voltage of cantilever beam 2 increases to 40.2 V while cantilever beam 1 drops to 11.0 V.
  Both simulation and experimental results identify *d* = 7 mm as the optimal distance for achieving distinct bimodal resonance peaks for both piezoelectric and electromagnetic voltages. Experimental voltages and resonance frequencies were slightly lower than the simulation results, potentially due to the actual stiffness of the cantilever beams being lower than the ideal values.
2. The electromagnetic damping force *F_cz_* increases as the load resistance *R_l_* decreases. In addition to damping the relative motion between the coils and permanent magnets at the ends of the two cantilever beams to generate electrical energy, *F_cz_* also enhances the intensity of the interaction force between the two beams and promotes their unification as a single structure.
  Simulation results indicate that as *F_cz_* increases, the resonance frequencies of the bimodal system shift toward higher frequencies; specifically, the first resonance point increases from 8.21 Hz to 8.33 Hz, and the second resonance point increases from 18.04 Hz to 18.48 Hz.
  Simulation calculations show that when *R_l_* decreases from ∞ to 10 Ω, the piezoelectric open-circuit voltage of cantilever beam 1 at the first resonance point increases from 23.9 V to 28.35 V, and that of cantilever beam 2 increases from 23.4 V to 26.7 V. At the second resonance point, the piezoelectric open-circuit voltage of cantilever beam 1 decreases from 15.6 V to 10.5 V, while that of cantilever beam 2 decreases from 18.1 V to 13.2 V. This trend was also verified in experiments, although the magnitudes of both the resonance frequency shifts and the piezoelectric voltage changes were smaller than the simulation results.
3. Experimental results indicate that in the piezoelectric module, the matched impedance of cantilever beam 1 at the first resonance point is approximately 10 MΩ with a maximum output power of 8.9 μW; at the second resonance point, the matched impedance is approximately 4 MΩ with an output power of 2.4 μW. For cantilever beam 2, the matched impedance at the first resonance point is 10 MΩ with an output power of 7.9 μW, and at the second resonance point, the matched impedance is 4 MΩ with an output power of 9.7 μW.
  In the electromagnetic module, the matched impedance at the first resonance point is 10 Ω with an output power of 0.39 W; at the second resonance point, the matched impedance is 15 Ω with an output power of 0.71 W.
  Compared to the magnetically coupled bistable M-shaped piezo-electromagnetic hybrid energy harvester proposed by Wang et al. [26], the coil in this design features only half the number of turns. However, due to the inclusion of an iron core and a larger wire diameter, the electromagnetic output power is significantly enhanced. By further implementing an array of multiple cantilevers equipped with iron-core coils and permanent magnets, a higher output power—potentially reaching the Watt-level—is anticipated.
  Regarding the piezoelectric film output, the use of small-volume single-layer PVDF piezoelectric films results in higher internal resistance and thus lower output power. When the material is replaced with PZT piezoelectric ceramic, the optimal matched impedance at the first resonance point (approximately 7.7 Hz) is about 450 kΩ. Under an amplitude of 2 mm, the output power reaches 0.37 mW, an improvement of approximately 120.5%. However, PZT materials are prone to fracture due to large deformations during cantilever beam resonance, making them unsuitable for the Bimodal device proposed in this study.

### 5.2. Application Scenarios

**Figure 18:**
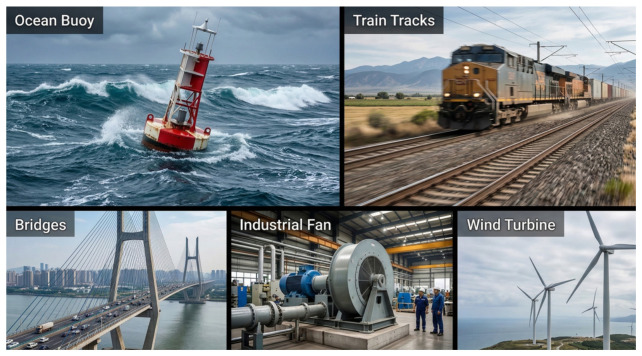
Common low-frequency vibration environments.

Common application scenarios are shown in Figure 18, and can be specifically divided into three categories:

1. Marine wave energy harvesting: Marine waves exhibit significant low-frequency and broadband characteristics, making it difficult for traditional resonant harvesters to adapt to the random variations in wave height and period. The piezoelectric–electromagnetic coupled device proposed in this paper utilizes the nonlinear coupling of dual-cantilever beams. By controlling parameters such as the length and thickness of the cantilever beams, as well as the gap *d* between the coil and the magnet, the device can achieve effective responses within the frequency bands corresponding to marine waves. It is highly suitable for self-powered systems in marine monitoring equipment such as buoys and submersible buoys. The device features a simple structure and a small volume, allowing it to be encapsulated within the buoy structure to prevent seawater corrosion. Furthermore, the bimodal resonance (or multi-resonance through array configurations) can cover the dominant frequency range of wave energy fluctuations, providing stable power support for maritime Internet of Things (IoT) nodes.
2. Rail transit and large machinery monitoring: Vibrations from train operations and industrial machinery are primarily concentrated within the 25 Hz range; however, significant frequency shifts occur during start-stop phases and variable speed conditions. The broadband response characteristics of this device are well-suited for the passive power supply of bearing health monitoring sensors and axle temperature detection nodes, thereby reducing wiring and maintenance costs. The adjustable damping characteristics of the electromagnetic coupling can further adapt to environments with different vibration intensities through the external load resistance *R_l_*, enhancing reliability under harsh operating conditions.
3. Building and infrastructure monitoring: Micro-vibrations (1–15 Hz) in large structures such as bridges, industrial fan and wind turbine towers contain continuous energy, but the frequency bands vary with environmental loads (e.g., wind loads, traffic flow). The multi-modal coupling mechanism of this device can be deployed in array networks. By utilizing parameter designs with different gaps *d* to achieve complementary frequency coverage, the system can provide distributed power for structural health monitoring (SHM) systems, serving as an alternative to traditional battery replacement schemes.

## Figures and Tables

**Table 1:** Key parameters of the device model.

Key Model Parameters	Value
Cylindrical magnet material,	N45 NdFeB
Residual magnetism *B_r_*	*B_r_* = 1.35 T
Radius of cylindrical magnet, *R_m_*	17 mm
Height of cylindrical magnet, *h_m_*	20 mm
Iron core radius, *R_d_*	12 mm
Iron core height, *h_d_*	20 mm
Outer radius of coil, *R_c_*_1_	17 mm
Inner radius of coil, *R_c_*_2_	12 mm
Wire radius of coil, *r_c_*	0.22 mm
Height of coil, *d_r_*	20 mm
Number of coil turns, *N*	700 turns
Thickness of piezoelectric film, *h_p_*	0.14 mm
Length of piezoelectric film, *L_p_*	28.2 mm
Width of piezoelectric film, *w_p_*	12.2 mm
Material of piezoelectric film	PVDF
Piezoelectric constant, *e*_31_	0.024 C/m^2^
Relative permittivity of piezoelectric material, *ε_s_*^33^	7.74
Width of cantilever beam, *W*	20 mm
Length of cantilever beam, *L*	135 mm
Thickness of Cantilever 1, *th*_1_	1 mm
Thickness of Cantilever 2, *th*_2_	1.4 mm
Initial horizontal gap between two cantilever beams, *d*	7 mm
Material of cantilever beam	65 Mn spring steel
Material of sleeve	PETG
Young’s modulus of piezoelectric cantilever, *E_y_*	206 GPa
Cantilever beam density, *ρ*	7850 kg/m^3^

**Table 2:** Comparison of the ideal simulation parameters, the actual experimental parameters, and the simulation results based on the actual parameters.

Resonant Frequencies	Ideal-Parameter Simulation Results (*th*_1_ = 1.0 mm, *th*_2_ = 1.4 mm, 206 GPa, etc.)	Experimental Results with Actual Parameters (*th*_1_ = 1.0 mm, *th*_2_ = 1.4 mm, 206 GPa, etc.)	Simulation Results with Actual Parameters (*th*_1_ = 1.0 mm, *th*_2_ = 1.4 mm, 206 GPa, etc.)
First resonant frequency	8.2 Hz	7.72 Hz	7.83 Hz
Second resonant frequency	18.0 Hz	15.74 Hz	15.92 Hz

**Table 3:** Comparison of dimensional parameters and output performance of different devices.

Reference Sources	External Excitation	Piezoelectric Module	Coil Parameters	Coupling Magnet Parameters	Maximum Power (mW)
Fan et al. (2018) [24]	a: 1.5 g f: ~7 Hz(*z*-axis), 33.4 Hz(*x*-axis)	M: PZT-5 H S: 30 × 7 × 0.25 MI: 230 kΩ	N: 200 D: 6 mm H: 8 mm WD: 0.13 mm R: 9 Ω	M: Nd-Fe-B D: 6 mm H: 8 mm MP: 7.95 × 10^5^ A/m	EEH: 1.42 PEH: 0.32
Zhang et al. (2023) [25]	a: 0.8 g f: ~12.5 Hz	M: K7520BS3 S: 75 × 20 × 0.43 MI: 220 kΩ	R: 175 Ω	M: Nd-Fe-B MP: 1 T	EEH: 15.2 PEH: 4.6
Wang et al. (2025) [26]	a: 1.4 g f: 8.4 Hz	M: PZT-5H S: 30 × 20 × 0.3 MI: 300 kΩ	N: 1300 R: 100 Ω	D: 20 mm H: 4.5 mm MP: 1 T	EEH: 1.24 PEH: 0.17
Xu et al. (2025) [27]	a: 1.0 g f: 15 Hz	M: PZT S: 30 × 10 × 0.4 MI: 1.6 MΩ	N: 1080 ID: 6 mm OD: 10 mm H: 10 mm R: 32 Ω	M: N52 D: 10 mm H: 16 mm MP: 10.8 × 10^5^ A/m	EEH: 324.49 PEH: 3.24
Zhang et al. (2024) [31]	a: 0.8 g f: 16.3 Hz	M: PZT-5 H S: 50 × 10 × 0.2 MI: 250 kΩ	N: 1800 ID: 10 mm OD: 21 mm H: 15 mm WD: 0.12 mm R: 150 Ω	M: N52 (Nd-Fe-B) D: 6 mm H: 5 mm MP: 7.9 × 10^5^ A/m	EEH: 28.6 PEH: 9.6
This work	a: 0.5 g f: ~8 Hz	M: PVDF S: 28.2 × 12.2 × 0.14 MI: 10 MΩ	N: 600 ID: 24 mm OD: 34 mm H: 20 mm WD: 0.35 mm R: 10 Ω	M: N45(Nd-Fe-B) D: 34 mm H: 20 mm MP: 1.35 T	EEH: 0.0168 (total) PEH: 387.5

## Data Availability

Data is contained within the article. The original contributions presented in this study are included in the article. Further inquiries can be directed to the corresponding author.

## References

[1] Ma D., Lan G., Hassan M., Hu W., Das S.K. (2019). Sensing, computing, and communications for energy harvesting IoTs: A survey. IEEE Commun. Surv. Tutor..

[2] Cai Z., Chen Q., Shi T., Zhu T., Chen K., Li Y. (2022). Battery-free wireless sensor networks: A comprehensive survey. IEEE Internet Things J..

[3] Qamar M.Z., Khalid Z., Shahid R., Tsoi W.C., Mishra Y.K., Kyaw A.K.K., Saeed M.A. (2024). Advancement in indoor energy harvesting through flexible perovskite photovoltaics for self-powered IoT applications. Nano Energy.

[4] Aldin H.N.S., Ghods M.R., Nayebipour F., Torshiz M.N. (2024). A comprehensive review of energy harvesting and routing strategies for IoT sensors sustainability and communication technology. Sens. Int..

[5] Zhang K., Liu S., An S., Zhou Y., Pu X. (2025). Towards high-efficiency self-powered wireless sensor networks: A systematic review of co-design of energy and signal. Nano Energy.

[6] Sun R., Zhou S., Cheng L. (2023). Ultra-low frequency vibration energy harvesting: Mechanisms, enhancement techniques, and scaling laws. Energy Convers. Manag..

[7] Jia N., Li Q., Li C., Du H., Gao X., Liu Y., Song K., Jin H., Ren K., Qiu C. (2024). A wireless ultrasound energy harvester based on flexible relaxor ferroelectric crystal composite arrays for implanted bio-electronics. Energy Environ. Sci..

[8] Zhao X., Yin X., Zhang Y., Xu K., Cai J. (2024). Study on impact pressing generator based on permanent magnet synchronous motor principles. J. Electron. Meas. Instrum..

[9] Chen L., Li C., Fang J. (2023). Design of a multi-direction piezoelectric and electromagnetic hybrid energy harvester used for ocean wave energy harvesting. Rev. Sci. Instrum..

[10] Joe H., Roh H., Cho H., Yu S.-C. (2017). Development of a flap-type mooring-less wave energy harvesting system for sensor buoy. Energy.

[11] Du Y. (2019). Broadband Vibrational Energy Harvesting: Research of Methods and Structural Design. Master’s Thesis.

[12] Zhou W., Du D., Cui Q., Yang Z., Lu C., Wang Y., He Q. (2022). Piezoelectric vibration energy harvester: Operating mode, excitation type and dynamics. Adv. Mech. Eng..

[13] Andò B., Baglio S., Bulsara A.R., Marletta V. (2014). A bistable buckled beam based approach for vibrational energy harvesting. Sens. Actuators A Phys..

[14] Lan C., Qin W. (2017). Enhancing ability of harvesting energy from random vibration by decreasing the potential barrier of bistable harvester. Mech. Syst. Signal Process..

[15] Zhou Z., Qin W., Du W., Zhu P., Liu Q. (2019). Improving energy harvesting from random excitation by nonlinear flexible bi-stable energy harvester with a variable potential energy function. Mech. Syst. Signal Process..

[16] Jackson N. (2020). PiezoMEMS nonlinear low acceleration energy harvester with an embedded permanent magnet. Micromachines.

[17] Zhang X., Chen L., Chen X., Zhu F., Guo Y. (2021). Time-domain dynamic characteristics analysis and experimental research of tri-stable piezoelectric energy harvester. Micromachines.

[18] Chen X., Zhang X., Chen L., Guo Y., Zhu F. (2021). A curve-shaped beam bistable piezoelectric energy harvester with variable potential well: Modeling and numerical simulation. Micromachines.

[19] Shao N., Chen Z., Wang X., Zhang C., Xu J., Xu X., Yan R. (2023). Modeling and analysis of magnetically coupled piezoelectric dual beam with an annular potential energy function for broadband vibration energy harvesting. Nonlinear Dyn..

[20] Atmeh M., Ibrahim A., Ramini A. (2023). Static and dynamic analysis of a bistable frequency up-converter piezoelectric energy harvester. Micromachines.

[21] Wu N., Fu J., Xiong C. (2023). A bio-inspired bistable piezoelectric structure for low-frequency energy harvesting applied to reduce stress concentration. Micromachines.

[22] Bradai S., Naifar S., Wolszczak P., Bieniaś J., Jakubczak P., Rysak A., Litak G., Kanoun O. (2025). Kinetic energy harvesting with a piezoelectric patch using a bistable laminate. Micromachines.

[23] Jin H., Tang B., Zhao C., Du S. (2024). The bistable piezoelectric energy harvester with magnetic attraction and repulsion between upper and lower beams. Chin. J. Sci. Instrum..

[24] Fan K., Liu S., Liu H., Zhu Y., Wang W., Zhang D. (2018). Scavenging energy from ultra-low frequency mechanical excitations through a bi-directional hybrid energy harvester. Appl. Energy.

[25] Zhang Y., Yang T., Du H., Zhou S. (2023). Wideband vibration isolation and energy harvesting based on a coupled piezoelectric-electromagnetic structure. Mech. Syst. Signal Process..

[26] Wang H., Zhao Q., Song R., Guo J., Chang W., Yang X., Zhang L. (2025). Design and performance study of low frequency magnetic coupling bistable piezoelectric and electromagnetic energy harvester. Energy.

[27] Xu J., Su X., Zhu B., Qian N., Chen X., Wen X., Yang Y., Leng Y. (2025). Performance evaluation and wireless sensing applications of an enhanced piezoelectric-electromagnetic hybrid energy harvester with bistable superposition mechanism. Mech. Syst. Signal Process..

[28] Zhang Y., Jin Y., Zhang Z. (2023). Dynamics of a tri-stable hybrid energy harvester under narrow-band random excitation. Int. J. Non-Linear Mech..

[29] Zhang T., Jin Y., Zhang Y., Sun P. (2026). Dynamics of a coupled quad-stable hybrid vibration energy harvesting system under dual-frequency excitation. Int. J. Non-Linear Mech..

[30] Yang B., He L., Ren H., Zhang L., Xu C., Wang Q. (2026). A piezoelectric-electromagnetic smart structure for wave energy harvesting. Eng. Struct..

[31] Zhang X., Cheng Y., Yang W., Pan J., Chen X., Xu H., Tian H., Zhang J. (2024). Theoretical analysis and experimental research of piezoelectric-electromagnetic hybrid vibration energy harvester. Smart Mater. Struct..

[32] Zhao L., Gong Y., Shen F., Peng Y., Xie S., Li Z. (2025). Diminishing potential well barrier in bi-stable energy harvesters by introducing symmetric stiffness. Thin-Walled Struct..

[33] Li Z., Liu Y., Yin P., Peng Y., Luo J., Xie S., Pu H. (2021). Constituting abrupt magnetic flux density change for power density improvement in electromagnetic energy harvesting. Int. J. Mech. Sci..

[34] Li Z., Yan Z., Luo J., Yang Z. (2019). Performance comparison of electromagnetic energy harvesters based on magnet arrays of alternating polarity and configuration. Energy Convers. Manag..

